# The landscape and driver potential of site-specific hotspots across cancer genomes

**DOI:** 10.1038/s41525-021-00197-6

**Published:** 2021-05-13

**Authors:** Randi Istrup Juul, Morten Muhlig Nielsen, Malene Juul, Lars Feuerbach, Jakob Skou Pedersen

**Affiliations:** 1 Department of Molecular Medicine, Aarhus University Hospital, Aarhus, Denmark; 2 Division of Applied Bioinformatics, German Cancer Research Center, Heidelberg, Germany; 3 Bioinformatics Research Centre, Aarhus University, Aarhus, Denmark

**Keywords:** Data processing, Cancer genomics

## Abstract

Large sets of whole cancer genomes make it possible to study mutation hotspots genome-wide. Here we detect, categorize, and characterize site-specific hotspots using 2279 whole cancer genomes from the Pan-Cancer Analysis of Whole Genomes project and provide a resource of annotated hotspots genome-wide. We investigate the excess of hotspots in both protein-coding and gene regulatory regions and develop measures of positive selection and functional impact for individual hotspots. Using cancer allele fractions, expression aberrations, mutational signatures, and a variety of genomic features, such as potential gain or loss of transcription factor binding sites, we annotate and prioritize all highly mutated hotspots. Genome-wide we find more high-frequency SNV and indel hotspots than expected given mutational background models. Protein-coding regions are generally enriched for SNV hotspots compared to other regions. Gene regulatory hotspots show enrichment of potential same-patient second-hit missense mutations, consistent with enrichment of hotspot driver mutations compared to singletons. For protein-coding regions, splice-sites, promoters, and enhancers, we see an excess of hotspots associated with cancer genes. Interestingly, missense hotspot mutations in tumor suppressors are associated with elevated expression, suggesting localized amino-acid changes with functional impact. For individual non-coding hotspots, only a small number show clear signs of positive selection, including known sites in the *TERT* promoter and the 5’ UTR of *TP53*. Most of the new candidates have few mutations and limited driver evidence. However, a hotspot in an enhancer of the oncogene *POU2AF1*, which may create a transcription factor binding site, presents multiple lines of driver-consistent evidence.

## Introduction

Mutations accumulate in human genomes throughout life. The majority are functionally neutral “passenger” mutations without effect on cell viability. Instead, accumulation of the few “driver” mutations that positively affect cell viability can cause cancer, as they enhance cells’ ability to proliferate, escape apoptosis, and eventually metastasize^1–3^. Driver mutations increase the relative fitness of cancer cells and increase in abundance through positive selection. In the past, driver discovery has focused on the identification of driver genes using whole-exome sequencing data. Whole-genome sequencing has enabled exploration of the 98% of the human genome that is non-coding. Surprisingly, this has only led to the discovery of a few well-confirmed non-coding cancer elements. The best-known example is the promoter of the oncogene *TERT*, which is involved in the elongation of the DNA telomere ends during replication^4,5^.

Mutations that activate oncogenes, and hereby give the cell growth advantages, are typically very specific and often recur in the same positions across patients, as they often modify active sites through a specific amino-acid change, as seen in *KRAS* and *BRAF* oncogenes^6–8^. Here, we call such individual recurrently mutated genomic positions for hotspots. In contrast, hotspots in tumor suppressors, which protect cells from abnormal growth, are not expected to the same degree, as the disruption of function can be obtained in many ways, such as gain-of-stop mutations, frameshift insertions/deletions (indels), and structural rearrangements^9^. The *TERT* promoter has two hotspots that both introduce a binding site for the ETS transcription factor when mutated and hereby increase transcription and cause overexpression of *TERT*^10–12^. Creation or modification of specific binding sites would often require specific changes of the DNA, potentially leading to hotspots if recurring across patients. Recently a non-coding hotspot of indels in the 5’ untranslated region (UTR) of *TP53*, which may affect splicing, has also been reported^13^.

The aim of this study is to detect, categorize, and characterize somatic site-specific single nucleotide variant (SNV) and indel hotspots genome-wide. The *TERT* promoter hotspots prove that non-coding drivers exist, we, therefore, set out to systematically evaluate the driver potential of hotspots outside protein-coding genes. We develop measures to evaluate signs of positive selection for individual hotspots applicable genome-wide. We catalog and analyze the driver potential of hotspots in a pan-cancer set of 2279 whole cancer genomes from the Pan-Cancer Analysis of Whole Genomes (PCAWG) project under the International Cancer Genome Consortium^14^. To our knowledge, the few previous genome-wide studies of hotspots have either been based on smaller patient cohorts^15–17^ or focused on mutational mechanisms^18^, whereas we focus on hotspot driver potential.

To assess the cancer driver potential of hotspots, measures such as gene expression and difference in cancer allele fractions (CAFs) between hotspots and other mutations can be used. Gene regulatory driver mutations are expected to affect expression. However, this effect may have been transient or dependent on other factors and may not be observable in the available tumor samples from patients, even if they have played a role during cancer progression. Driver mutations are expected to have high CAFs compared to passenger mutations, as key driver mutations are expected primarily early in tumor evolution^1^, whereas passengers are expected throughout cancer evolution and thus will more often be subclonal^1,2^.

In addition to recurrent positive selection, the heterogeneity of the mutational processes in cancer may also cause hotspots. Mutational processes often prefer a specific context leading to highly localized accumulation of mutations and potentially hotspots^19–21^. Individual processes have distinct mutational patterns, typically represented by mutational signatures^20–22^. Both external factors, such as exposure to carcinogens, like smoke^23^ or UV radiation^24,25^, and internal factors like defects in repair genes or pathways, such as the BRCA genes^26,27^, can lead to a distinct mutational pattern. Mutational signatures are usually described using the nucleotide exchange and its +/−1 base pair (bp) context, but examples of longer sequence preferences exist, including a six-bp-long context for a UV mutational mechanism^28^. Many other complex sequence-dependencies and context-dependencies undoubtedly exist, uncaptured by current mutational signatures, which may contribute to the formation of hotspots.

Technical artifacts may result in false mutation calls, potentially recurrent across patients. In particular, sequencing errors are known to accumulate in certain sequence contexts^29^. Similarly, mapping errors will often be recurrent, as caused by the structure of the genome and potentially shared segregating polymorphism, including structural variants^30^.

Here we identify a total of 722,924 somatic site-specific hotspots. We group hotspots according to their genomic regions and relation to cancer genes. In our analysis of hotspot driver potential, we evaluate the excess of cancer genes among hotspots combined with an analysis of CAFs, expression, and for SNV hotspots, signature contributions. Overall, we find more hotspots than expected given models of the mutational background, and the protein-coding regions have a general enrichment of SNV hotspots compared to all other regions. Furthermore, we find an excess of hotspots associated with cancer genes compared to other genes in various regions, including enhancers, where an SNV hotspot in an enhancer for the oncogene *POU2AF1* comes up with signs of positive selection in multiple of our analyses. For a few regions, we see expression aberrations of hotspots compared to wild-type, and, interestingly, we observe a rise in the expression level among missense mutations in tumor suppressors.

## Results

### Hotspots are present across the genome

We analyzed the set of 2279 whole cancer genomes from PCAWG. Sample collection, variant calling, and curation were done by the consortium^14^. In total, the dataset encompasses 30,171,668 SNVs, 1,477,513 deletions and 728,918 insertions. In the following, we first identified somatic site-specific SNV-, deletion-, and insertion hotspots as genomic positions or regions that were recurrently mutated across patients. The hotspots were then grouped and analyzed based on functional region and recurrence among patients. We thereafter characterized hotspots by the comprising mutations’ association with potential gain or loss of transcription factor binding sites (TFBSs), high CAFs, gene expression, and, for SNVs, contributions of specific mutational signatures. We also characterized hotspots based on their location in different genomic regions, such as homopolymer runs, repeat regions, or duplicated genomic regions. We finally evaluated the evidence that recurrent positive selection has caused hotspots, both by evaluating enrichments among cancer genes and on a case-by-case basis (see “Methods” section).

**Fig. 1:**
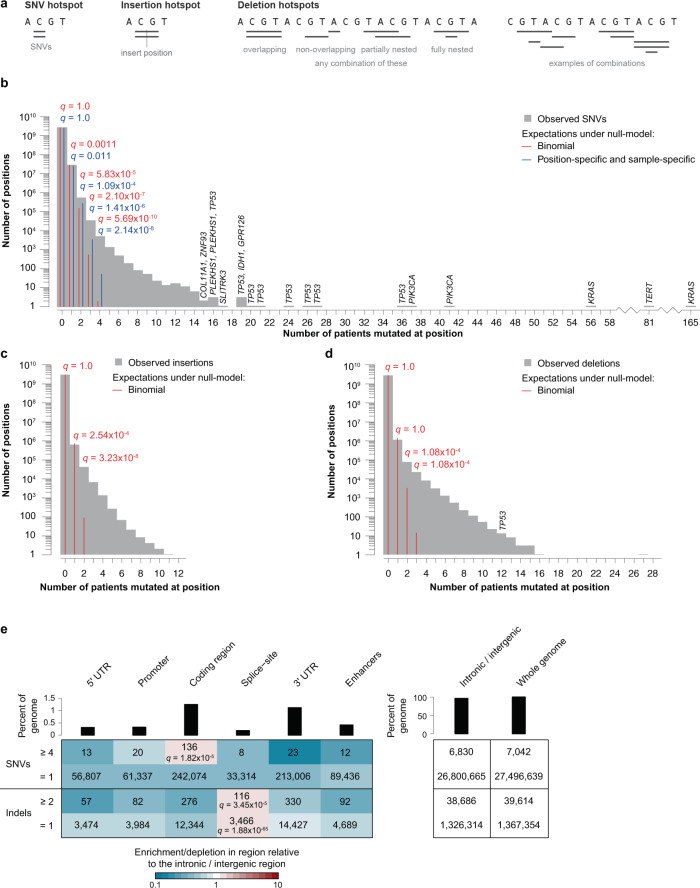
Overview of detected site-specific hotspots. **a**. Schematic definition of SNV-, insertion-, and deletion hotspots. **b–d** Bar-charts showing the number of hotspots with a specific number of mutations for SNVs (b), insertions (c), and deletions (d) with expectations under null hypotheses; exact *q*-values are reported when null models predict at least one hotspot; remaining *q*-values are reported in Supplementary Table 1. **e**. Heatmap of hotspot enrichment/depletion across various genomic regions; bars show the genomic extent of each region; for the protein-coding and gene regulatory regions *q* = 1 when not stated explicitly.

We identified somatic hotspots genome-wide for all three mutation types. The genomic extent and practical definition of hotspots depend on the type of mutation: SNV hotspots always span a single genomic position; insertion hotspots always fall between two consecutive genomic positions; whereas deletion hotspots may have varying extent defined as the overall span of the individual included deletions (Fig. 1a). In addition to the quality assessment of the underlying mutation calls by the PCAWG consortium, we further included a conservative quality filter for the high-frequency SNV hotspots (see “Methods” section).

Overall, we identified 722,924 hotspots; 566,760 of which were SNV hotspots, 46,748 were insertion hotspots, and 109,416 were deletion hotspots. The majority (98.10%) of hotspots were located in the intronic and intergenic regions, 0.61% in protein-coding regions (3989 SNV hotspots, 101 insertion hotspots, and 346 deletion hotspots), and 1.29% in gene regulatory regions (6558 SNV hotspots, 629 insertion hotspots, and 2113 deletion hotspots).

### Highly mutated hotspots are unexpected

The expected number of hotspots with a given number of mutations can be evaluated using statistical models of the distribution of mutations across the genome and patients. For SNVs, we use two different models. The first model simply assumes the same average mutation probability for each patient and genomic position (binomial model; see “Methods” section). The second model uses a rich set of genomic features to predict the mutation probability at each genomic position of every included sample^31,32^. It captures known sources of variation in the mutation rate, including patient-specific mutational signatures by considering the trinucleotide context of each mutation, genomic region, gene expression, phyloP positional conservation scores, and replication timing (position-specific and sample-specific model; see “Methods” section). The positional conservation scores capture the effects of both selection and site-specific mutation rate through species evolution. None of the models predicted any SNV hotspots with more than four mutations across patients with a false discovery rate (see “Methods” section) below 0.001% (*q* < 0.00001; Fig. 1b; Supplementary Table 1). This contrasts our observation of 2162 SNV hotspots with at least five mutations and a general excess of hotspots.

For insertion and deletion hotspots, we similarly evaluate the genome-wide expectancies using binomial models. We predict no insertion hotspots involving more than two patients, and no deletion hotspots with more than three patients with a false discovery rate below 0.001% (*q* < 0.00001; Fig. 1c, d).

For the final analysis, we focus on hotspots that are unexpected. For SNVs we use the position-specific and sample-specific model, and for insertions and deletions, we simply assume uniformity using the binomial model. In both cases, we only include hotspots with a false discovery rate of less than 10%. This led to the inclusion of SNV hotspots with four or more mutations (Supplementary Data 1; SNV hotspots with 2–3 mutations can be found in Supplementary Data 2), and insertion and deletion hotspots with two or more mutations (Supplementary Data 3). For the remaining analysis, we group insertion and deletion hotspots together as indel hotspots.

### Hotspots are unevenly distributed in different functional regions of the genome

We divided the hotspots based on their overlap with functional regions (protein-coding, splice-sites, 5’ and 3’ UTRs, promoters, enhancers, and intronic/intergenic regions). We then assessed the distribution of the hotspots across these regions and evaluated their enrichment relative to the intronic/intergenic region, which spans the majority of the genome (96.39%; Fig. 1e).

Of the 7042 highly mutated SNV hotspots (*n* ≥ 4), small fractions overlap protein-coding (1.93%) and gene regulatory regions (1.08%). Only protein-coding regions were slightly enriched for hotspots (1.53x; *q* = 1.82 × 10^−5^), while the gene regulatory regions had varying degrees of hotspot depletion (0.29–0.87x; Fig. 1e). When restricting to hotspots recurrently mutated in at least five patients (*n* = 2162), we observe further enrichment among protein-coding regions (3.25x; *q* = 6.15 × 10^−19^), and non-significant enrichments among promoters (1.75x; *q* = 0.287), 5’ UTRs (1.51x; *q* = 0.667), and splice-sites (1.27x; *q* = 1; Supplementary Fig. 1). To evaluate whether background mutational processes could have created these patterns, we also checked the regional distribution and relative enrichments of singleton mutations. These were generally depleted in both protein-coding and gene regulatory regions (range: 0.64–0.77x). We, therefore, conclude that the relative enrichment of hotspots in some regions is unlikely to be explained solely by the mutational processes that govern the distribution of singleton mutations. Our set of highly mutated SNV hotspots include 80 known driver positions. We use the patterns and range of values in these hotspots as an indication of selection.

We saw large depletions of indel hotspots (*n* ≥ 2) across all our functional regions (0.23–0.84x) except splice-sites (1.63x; *q* = 1.02 × 10^−22^; Supplementary Fig. 1). This hotspot depletion in functional regions was partly explained by an enrichment of intronic and intergenic hotspots in homopolymer runs, likely caused by polymerase slippage during replication of repetitive regions^33^, both as part of the naturally occurring mutational process and during sequencing. A recent study investigated the mutational landscape of microsatellites, including homopolymer runs, using the PCAWG dataset^34^. They found an elevated mutation rate for long repetitive regions (>9 bp) and found that the majority of mutated microsatellites were homopolymer runs of A/T nucleotides. We found that 18% of indel singletons and as much as 75% of indel hotspots are located in homopolymer runs, and together with the findings of Fujimoto et al.^34^, this highlights the need for filtering indel hotspots (see “Methods” section). In subsequent indel analysis, we eliminated homopolymer runs and overlapping indels as they dominate and dilute signals, reducing the total number of indel hotspots to 39,614.

After removal of indel hotspots at homopolymer runs, indel hotspots were still depleted across all regions (0.45–0.73x) except splice-sites (1.56x; *q* = 3.45 × 10^−5^; Fig. 1e). This pattern mimics the relative depletion of indel singletons, which similarly show enrichment in splice-sites (1.36x; *q* = 1.88 × 10^−68^) and depletion in all other regions (0.71–0.93x). This suggests that the majority of regulatory indel hotspots may be explained by the background mutational processes, though the enrichment in splice-sites is likely driven by selection^35^.

We evaluated whether patients with mutations in gene regulatory hotspots had missense mutations affecting the same gene. We expect to see such missense mutations in tumor suppressors, where double hits are required to cause inactivation, as well as in oncogenes, where amino acid altering mutations can lead to activation. Therefore such missense mutations may indicate a functional impact of the hotspot. Of the 735 gene regulatory hotspots (76 SNV hotspots, *n* ≥ 4; 659 indel hotspots, *n* ≥ 2), 89 hotspots had additional missense mutations in at least one of the involved patients. Seven of these hotspots had missense mutations in multiple patients. However, three of the hotspots are caused by POLE deficient hyper-mutators and another hotspot is affected by a focal amplification; the remaining three lack clear causes (Supplementary Note). Interestingly, the gene regulatory hotspots are enriched (81x) for these missense mutations compared to the fraction of gene regulatory singletons with potential same-patient second-hit missense mutations (702 out of 483,195; *p* < 2.2 × 10^−16^).

### Known cancer genes harbor high-frequency hotspots

**Fig. 2:**
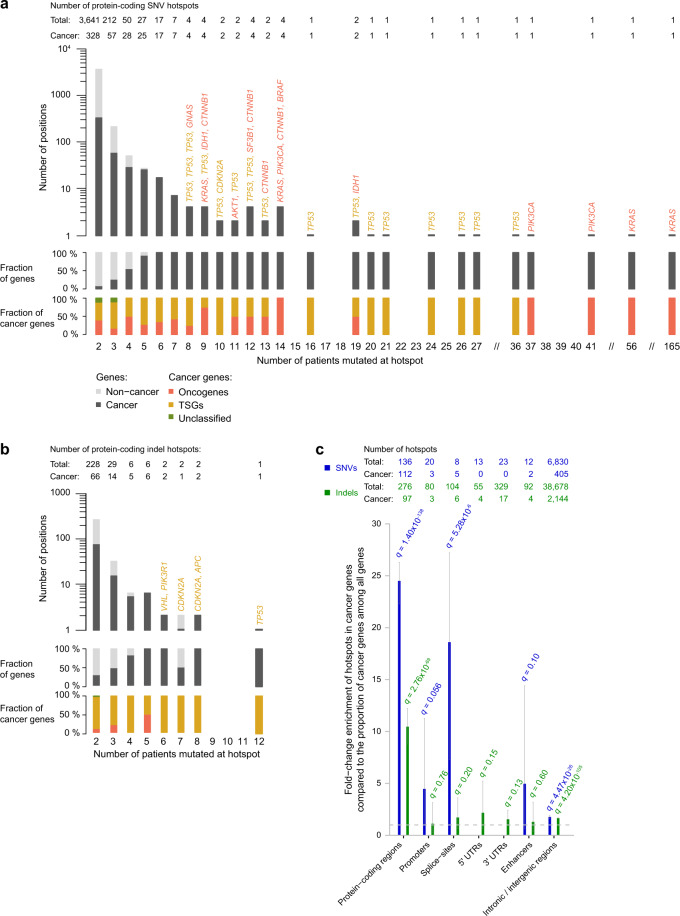
Cancer gene enrichments. **a**–**b** Bar-charts of protein-coding SNV (a) and indel (b) hotspots; numbers above bars indicate the total number of hotspots and hotspots in cancer genes; gene names are shown for hotspots in oncogenes and tumor suppressors with more than seven (SNVs)/five (indels) mutations; the middle bar-plot show the fractions of cancer genes among the hotspots; the lower bar-plot show the fraction of oncogenes, tumor suppressors, and unclassified cancer genes in the total amount of cancer genes. The remaining regions are included as Supplementary Fig. 2 for SNVs and Supplementary Fig. 3 for indels. **c** Fold-change enrichment of hotspots in known cancer genes relative to the overall proportion of cancer genes among all genes (699/20,805); numbers above bars are the total number and number of cancer hotspots in each region; error bars are Clopper-Pearson 95% confidence interval approximations. TSGs: Tumor suppressor genes.

The most frequently mutated SNV hotspot had 165 mutations across the 2179 patients (7.6%; Fig. 1b). Another ten hotspots had mutations in 20 or more patients, and all of these frequently mutated hotspots are known cancer driver mutations. A single of these, a hotspot with 81 mutations, is located in one of the known *TERT* promoter hotspot positions, while the others are in protein-coding regions. Six of these hotspots were in the tumor suppressor *TP53*, which is highly mutated across cancer types (Fig. 2a)^36^. The remaining three high-frequency hotspots were in oncogenes.

Compared to the SNVs, protein-coding indel hotspots did not reach the same extreme frequencies. The hotspot with the highest frequency was located in the intronic/intergenic region and had 27 deletions (Fig. 1c; d). The protein-coding indel hotspot with the highest frequency was in *TP53* and consisted of 12 deletions (Fig. 2b). Not surprisingly, *TP53* was the most frequently mutated cancer gene overall. We observed 103 SNV and 26 indel hotspots across its coding region, making it the gene with far the most hotspots as well. Furthermore, regulatory regions for *TP53* were also heavily affected by hotspot mutations, as we saw 12 SNV splice-site hotspots, a single indel splice-site hotspot, and a single indel 5’ UTR hotspot^13^.

We systematically went through all hotspots of high frequency (>10 mutations; *n* = 72), with all but six being SNV hotspots. Of the 31 hotspots associated with cancer genes, most are in the protein-coding (*n* = 26) or gene regulatory regions (*n* = 3; described above), with two found in more distal intergenic regions associated with *BCL11A* and *KIAA1598* (Fig. 2a; b; Supplementary Figs. 2; 3). None of the 41 hotspots associated with non-cancer genes are in the protein-coding region and only a few are in gene regulatory regions (*n* = 4). Both the remaining two hotspots associated with cancer genes and the hotspots associated with non-cancer genes either had no clear explanation, were caused by specific mutational mechanisms, or were likely artifactual (Supplementary Note).

### Limited hotspot enrichment in regulatory regions of known cancer genes

We sought to evaluate whether the positive selection and the presence of driver mutations could explain some of the uneven hotspot distribution among functional regions. Protein-coding driver mutations are enriched among already known cancer genes, as expected^37^. If non-coding driver mutations in regulatory regions are present, we similarly expect they would affect cancer genes more often than other genes, as exemplified by the presence of non-coding driver hotspots in the *TERT* promoter. As an initial proxy for the presence of drivers, we, therefore, evaluated whether a surprising fraction of hotspots are associated with known cancer genes for individual functional regions.

We first evaluated the enrichment of hotspots affecting protein-coding or gene regulatory regions of cancer genes under the assumption that a random mutation is equally likely to hit a base in a cancer gene as in any other gene. For our list of cancer genes, we use a set of 699 curated Cancer Gene Census genes maintained by COSMIC^38^. The 7042 highly mutated SNV hotspots were enriched in the protein-coding regions of cancer genes (24.5x; *q* = 1.40 × 10^−138^) and in their promoters (4.5x; *q* = 0.056), splice-sites (18.6x; *q* = 5.28 × 10^−6^), and enhancers (5.0x; *q* = 0.10; Fig. 2c). Indel hotspots were strongly enriched in the protein-coding region of cancer genes (10.5x; *q* = 2.76×10^−69^; Fig. 2c), with limited non-significant levels of enrichment in regulatory regions (1.1–2.2x). To ensure that size and mutation rate differences among genes did not explain this, we also evaluated the relative enrichment of hotspots to singleton mutations in cancer genes (see “Methods” section). As drivers may also elevate the fraction of cancer genes among singletons, thereby lowering the relative enrichment for hotspots, this measure is expected to be more conservative. We saw an enrichment of SNV hotspots in the same genomic regions, though not as pronounced, and for some regions non-significant (13.7x; *q* = 1.06 × 10^−110^; 3.1x; *q* = 0.21; 10.4x; *q* = 1.52 × 10^−4^; 2.9x; *q* = 0.35; Supplementary Fig. 4). The limited enrichment for indel hotspots in regulatory regions of cancer genes was lost when we compared to singleton indels in cancer genes (0.8–1.2x; Supplementary Fig. 4).

For protein-coding SNV hotspots with more than just a few mutations, cancer genes dominate (Fig. 2a). All SNV hotspots with six or more mutations (*n* = 59), were found in cancer genes. Besides many hotspots in *TP53* (*n* = 27), various oncogenes are among these. As expected, the vast majority of protein-coding indel hotspots in cancer genes affect tumor suppressors (83 of 97). Again with *TP53* being the most frequently affected (26 of 83) (Fig. 2b).

### Hotspots that may affect transcription factor binding sites

We investigated if SNVs or indels at hotspots could cause either gain or loss of TFBSs. We focus this analysis on protein-coding and gene regulatory hotspots where we know that binding is possible by selecting the hotspots that overlap ENCODE transcription factor binding peaks (TFP; see “Methods” section). This includes 212 SNV hotspots and 936 indel hotspots.

For SNVs, 36 of the 212 highly mutated hotspots overlapped TFPs. Of these, seven overlapped individual transcription factor motifs and five of these were found to potentially cause gain or loss (see “Methods” section). Only one of these is previously reported, namely one of the known *TERT* promoter hotspots, where the mutations result in the formation of an ETS TFBS^10–12^. The other four include: A hotspot in a *POU2AF1* enhancer could create a TFBS for three NF-E2-like transcription factors. This hotspot is also identified in other types of analyses and is discussed further below. A hotspot in the 3’ UTR of *PI15* is located in a conserved element and potentially causes disruption of a TFBS for CTCF-like factors. The other two hotspots are found in a promoter of *NRD1* and in the 5’ UTR of *C1orf159*, and they are likely caused by mutational mechanisms, as they have high contributions of esophagus-related signatures and APOBEC signatures, respectively. Furthermore, the *C1orf159* hotspot is located in an optimal APOBEC3A binding site, providing further support for it being caused by a mutational mechanism. Additional details on individual cases, as well as a description of our signature analysis based on hotspot sets, can be found in the Supplementary Note.

For indels, 125 of the 936 hotspots overlapped TFPs. Twelve of these overlapped individual transcription factor motifs and two of these could potentially cause TFBS gain or loss. One is an insertion hotspot in the protein-coding region of *VHL*, which potentially interrupts a binding motif for ZBTB6, a BTB-ZF-like transcription factor. The other is a deletion hotspot in the protein-coding region of *RASAL2*, which potentially creates a binding motif for the STAT6 transcription factor (Supplementary Note).

### Some gene regulatory hotspots may be mutated early in cancer development

Mutations that happen early in the cancer development will have high CAF values as most clones would harbor the mutation^39^. CAF is a normalized measure of the variant allele fraction that estimates the tumor read fraction carrying the variant (see “Methods” section). For each mutation in each patient, we calculated the CAF and a more robust measure called ΔCAF that evaluates the relative change in CAF compared to CAF of surrounding mutations, which accounts for high CAFs caused by copy number decrease of larger regions. We use *z*-score normalization on all ΔCAF values. A high *z*-score suggests that the mutation was an early event in the development of cancer.

**Fig. 3:**
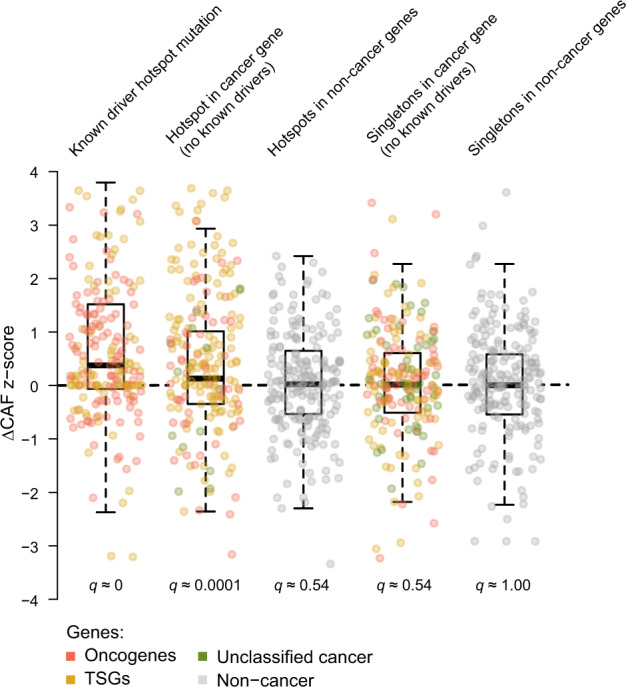
Boxplot of ΔCAF z-score distributions. One boxplot for each group of SNV hotspots with four or more mutations and singletons in the protein-coding region; the first boxplot is known driver hotspots; the second is hotspots in cancer genes excluding driver positions; the third is hotspots in other genes; the fourth is all singletons in cancer genes excluding driver positions; the last is all singletons in other genes; dots are 200 randomly sampled hotspots/singletons for each boxplot; the centerline of each box is the median of the distribution; the lower bound of the box is the first quartile (Q1); the upper bound of the box is the third quartile (Q3); the lower whisker shows the minimum value greater than Q1 − 1.5x IQR; the upper whisker shows the maximum value smaller than Q3 + 1.5x IQR. IQR: interquartile range (Q3–Q1); CAF: cancer allele fraction; TSGs: tumor suppressor genes.

We tested if the median *z*-score was high compared to a null distribution for known driver hotspots, as well as other protein-coding hotspots in both cancer and other genes, and protein-coding singletons in both cancer and other genes (overall median *z*-score = 0.004; see “Methods” section). Known driver hotspots generally seem to be early events in cancer development (median *z*-score = 0.38; *q* ≈ 0.0). Other protein-coding hotspots in cancer genes also seem to be early events, though not as early as the known drivers (median *z*-score = 0.13; *q* ≈ 0.0001). The remaining regions did not show significant ΔCAF *z*-scores (Fig. 3). To annotate individual hotspots as potentially early, we define a threshold for high ΔCAF z-scores as the 90th percentile of the *z*-score distribution for protein-coding hotspots in cancer genes (threshold of 2.05). To select hotspots from small sets, we use the above-median *z*-score (>0.13) as a cutoff.

**Fig. 4:**
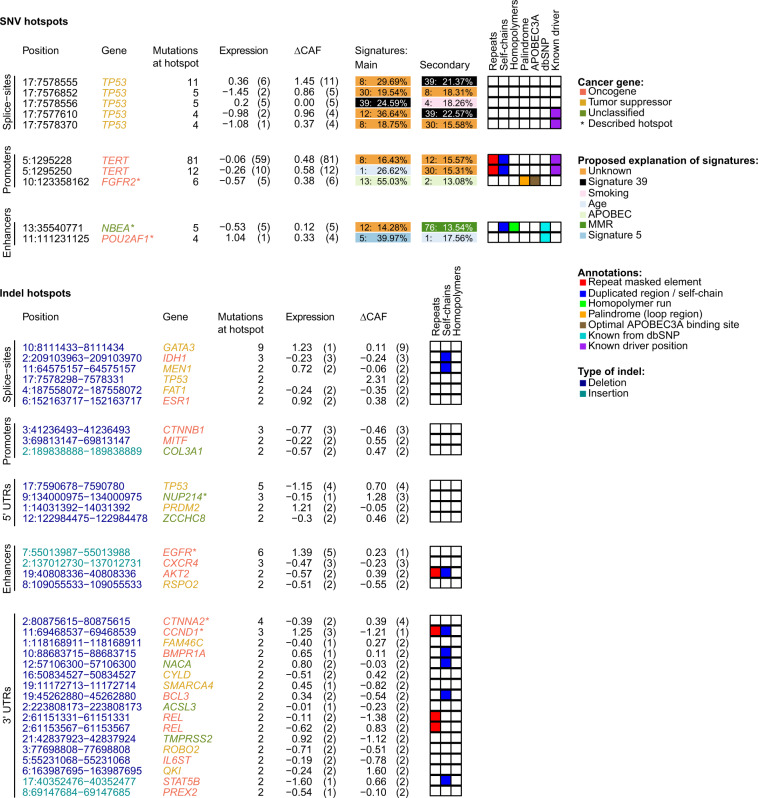
Description of gene-regulatory hotspots related to cancer genes. Annotation of position, gene, number of mutations at a hotspot, expression *z*-score, ΔCAF *z*-score and three (indels)/six (SNVs) genomic features; for SNVs the annotations further include the top-2 signatures; includes all gene-regulatory indels with two or more mutations and SNVs with four or more mutations associated with cancer genes; positions are given according to the hg19 reference genome. MMR: mismatch repair.

Across the highly mutated SNV hotspots (*n* = 7042), two-fifths had above-median z-scores (~40%), while only five had high z-scores (0.07%). These hotspots were either in the protein-coding region (2 of 5) or the intronic/intergenic region (3 of 5). Ten of the highly mutated gene regulatory SNV hotspots (*n* = 76) were associated with cancer genes, whereof eight had above-median *z*-scores (Fig. 4). These include five *TP53* splice-site hotspots, three promoter hotspots, including the two *TERT* promoter hotspots and an *FGFR2* promoter hotspot, and two enhancer hotspots in enhancers for *NBEA* and *POU2AF1*.

Across the indel hotspots, one-third (~36%) had above-median *z*-scores, whereas only a small fraction had high *z*-scores (0.11%). Among the gene regulatory indel hotspots (*n* = 659), 34 were associated with cancer genes (Fig. 4). These include 15 hotspots with above-median *z*-scores, hereof a single *TP53* splice-site hotspot with a high *z*-score, and among others, two hotspots in the 5’ UTRs of *TP53* and *NUP214*, a hotspot in an enhancer for *EGFR*, and a hotspot in the 3’ UTR for *CTNNA2*. We describe the few gene regulatory SNV and indel hotspots associated with cancer genes with an above-median *z*-score (marked with * in Fig. 4) later or in the Supplementary Note.

### Elevated expression levels among tumor suppressors with missense mutations

Next, we evaluated whether hotspots associate with aberrant expression levels compared to wild-types. Cancer gene hotspots were further divided into oncogenes, tumor suppressors, and unclassified genes, as our expectations are different for oncogenes and tumor suppressors. For hotspots in protein-coding regions, we stratified mutations further into missense and nonsense mutations, and frameshift and in-frame indels, based on the variant classification. We have gene expression data for a large subset of patients (~45%) in our dataset. To enable comparison across cancer types, we normalize the expression values per gene and per cancer type, and represent them as *z*-scores (see “Methods” section).

Due to nonsense-mediated decay, we expect decreased expression levels for nonsense mutations and frameshift indels. On the contrary, missense mutations and in-frame indels are not affected by this mechanism, so they do not necessarily affect the mRNA level. We expect mainly missense mutations in oncogenes, as specific amino acid changes are typically needed to activate these genes^6–8^. Even though the missense mutations in oncogenes may not directly lead to increased expression levels, we expect increased expression levels in oncogenes potentially caused by epigenetics or the selection of regulatory mutations.

**Fig. 5:**
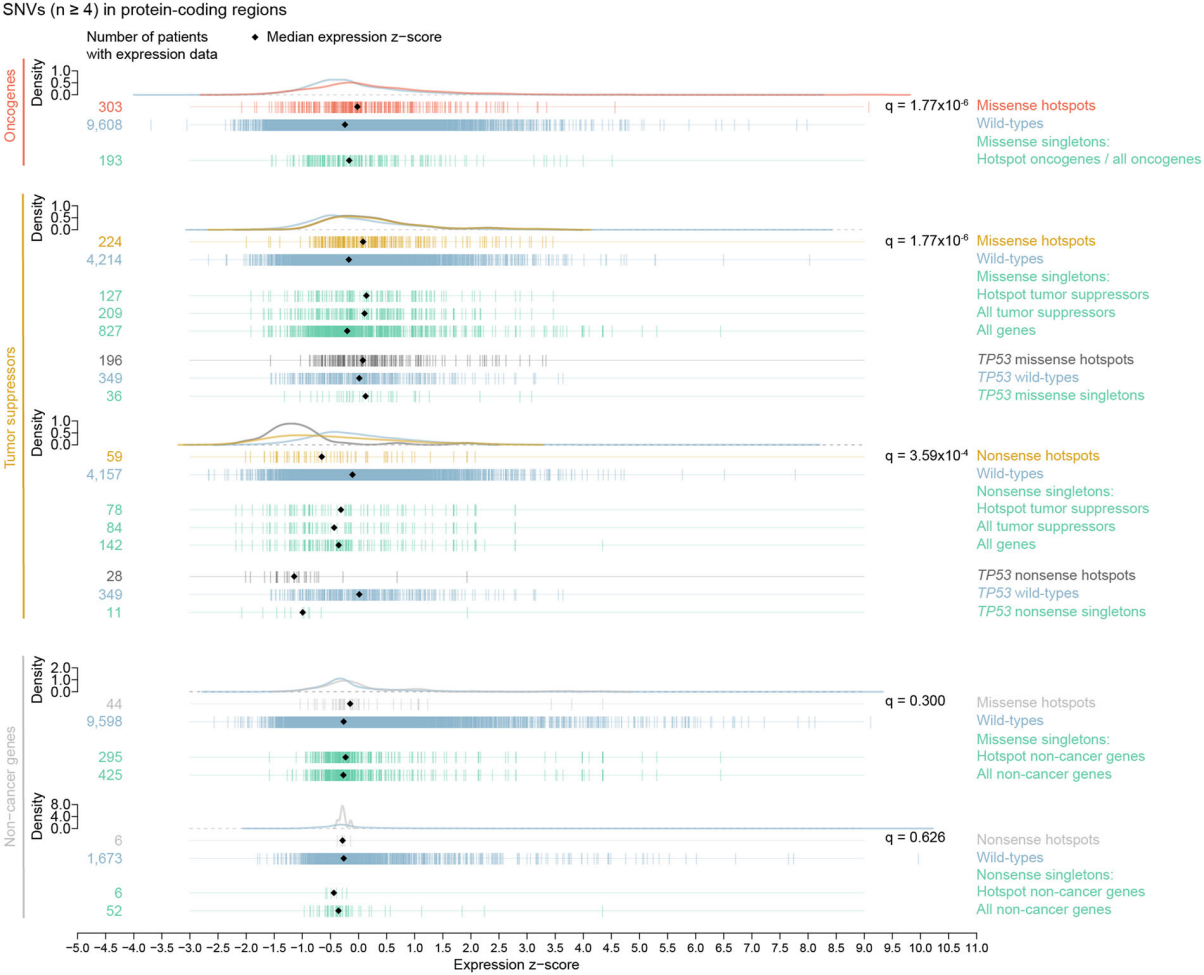
Distribution of expression *z*-scores for patients with hotspot mutations. Expression distributions for SNV hotspots with four or more mutations in protein-coding regions of oncogenes, tumor suppressors, and non-cancer genes divided into missense and nonsense mutations; wild-type expression for genes included in hotspot sets are shown directly below each set; singleton expression is shown for subsets of genes below each set; a subset of all *TP53* hotspot mutations are shown below the tumor suppressors.

Across highly mutated protein-coding SNV hotspots, we observed expression aberrations for hotspots with missense mutations in oncogenes (shift = 0.25; *q* = 1.77 × 10^−6^) and hotspots with nonsense mutations in tumor suppressors (shift = −0.51; *q* = 3.59 × 10^−4^) when compared to wild-type expression in the same genes, as expected. Interestingly, we also observed expression aberrations for hotspots with missense mutations in tumor suppressors (shift = 0.28; *q* = 1.77 × 10^−6^; Fig. 5; Supplementary Table 2; “Methods” section). These expression aberrations could potentially be caused by selection, by some tumor suppressors being bivalent with oncogenic properties, or by feedback mechanisms, where the aberrant protein function leads to increased gene expression levels. If the latter is the case, we would expect a similar rise in expression levels among patients with singleton missense mutations. We do see a similar pattern for the missense singletons both for tumor suppressors in general and for the subset of tumor suppressors harboring hotspots (shifts = 0.21; Fig. 5). Neither missense (shift = 0.10; *q* = 0.300) nor nonsense mutations in non-cancer genes (shift = −0.014; *q* = 0.626) were associated with significant aberrations. For the protein-coding indel hotspots, only frameshift indels in tumor suppressors (shift = −0.36; *q* = 1.89 × 10^−4^) and non-cancer genes (shift = −0.17; *q* = 4.17 × 10^−4^) had significant aberrations (Supplementary Fig. 5).

### Few individual hotspots show association with expression aberrations

Individual hotspots may have extreme expression levels and be associated with expression aberrations, even when the larger group of hotspots show no overall effect on expression. Therefore, we evaluate individual hotspots or smaller groups of hotspots associated with the same gene. The individual hotspots with the largest expression aberrations are already known to be associated with cancer (Supplementary Fig. 6), including the two promoter SNV driver hotspots in *TERT*^4,5^, multiple splice-site SNV hotspots in *TP53*^40^, and a 5’ UTR indel hotspot in *TP53*^13^.

Overall, the mutations in the *TERT* hotspots are associated with a small rise in median expression compared to wild-type. These hotspots are mutated across multiple cancer types, and it is well-known that mutations at these positions alter gene expression in thyroid cancer^12^. When stratified by cancer type, we see large expression aberrations in both thyroid cancer and two brain cancers (oligodendroglioma and glioblastoma; Supplementary Fig. 6). Patients with deletions in the 5’ UTR indel hotspot in *TP53* had much lower expression than wild-types, and, in contrast, the general expression level of tumor suppressors with 5’ UTR indels was elevated (Supplementary Fig. 6). Besides these known hotspots, we identified two other indel hotspots associated with cancer genes with large expression aberrations. These are in an enhancer of *EGFR* and in the 3’ UTR of *CCND1* and are likely artifacts (Fig. 4; Supplementary Fig. 6; Supplementary Note).

### Candidate driver hotspot in *POU2AF1* enhancer

**Fig. 6:**
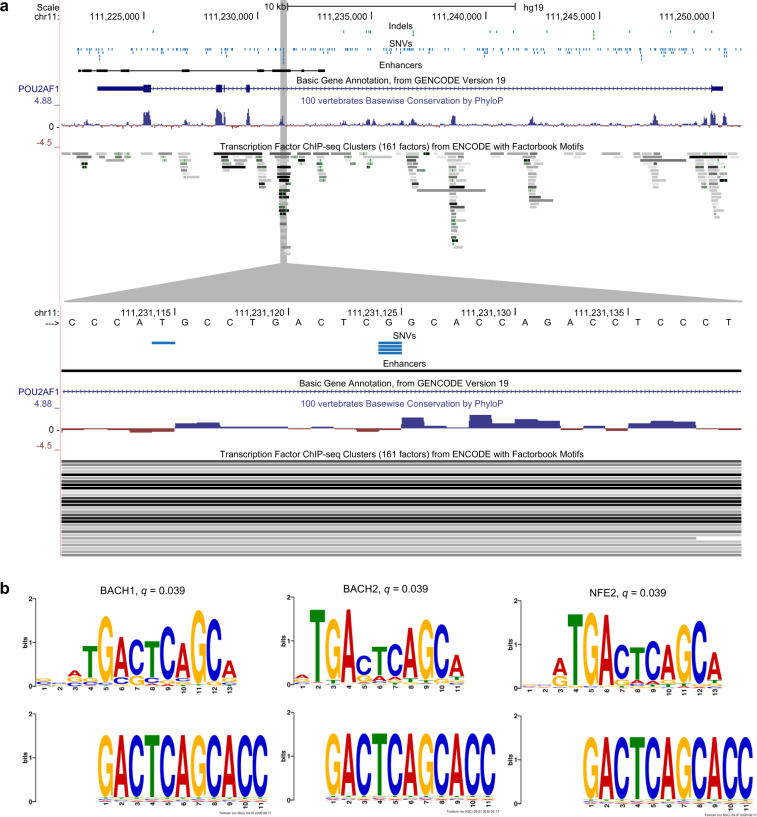
Genomic region around *POU2AF1*. **a** Genomic features in the area around the *POU2AF1* gene including locations of SNVs and indels in the PCAWG dataset, location of enhancers, conservation, and ENCODE transcription factor peaks; the upper panel show the full region; the lower panel show a zoom-in on the hotspot in the *POU2AF1* enhancer (made with the UCSC GenomeBrowser). Genomic coordinates are given according to the hg19 reference genome. **b** Logo-plots for the transcription factor binding motifs for BACH1, BACH2, and NFE2 (made with TomTom^49^).

In multiple of our individual analyses, the findings suggest that an enhancer hotspot associated with the oncogene *POU2AF1* may be under positive selection: the hotspot come up as a top result in the TFBS analysis; it contributes to the enrichment of cancer genes among enhancer hotspots; it has an above-median ΔCAF *z*-score; and based on contributions of mutational signatures, we do not believe that background processes caused these mutations. The hotspot has four mutations across patients, three in liver hepatocellular carcinoma and one in head and neck squamous cell carcinoma. It is located in an intronic enhancer 44 kilobases downstream of the transcription start site of *POU2AF1* (Fig. 6a). The three liver cancer patients had G > C mutations, whereas the head and neck cancer patients had a G > A mutation. The G > A mutation creates transcription factor binding motifs for the three NF-E2-like transcription factors, BACH1, BACH2, and NFE2 (Fig. 6b). Two of the ΔCAF *z*-scores were around the 65th percentile (0.59; 0.54) and the other two were around the 45th and 35th percentile, respectively (0.05; −0.18). Among its top-3 signatures we find signatures 5 (40.0%), 1 (17.6%), and 39 (17.0%), none of which indicates that this hotspot should be caused by background mutational processes. We could not perform expression analysis due to a lack of data. In conclusion, the potential gain of TFBS and the few higher ΔCAF *z*-scores suggest that the *POU2AF1* enhancer hotspot may be under positive selection, but further evidence is needed to establish this. To validate this hotspot, we searched for mutations in the COSMIC database (v. 92)^41^, where we found two confirmed-somatic G > C mutations in liver cancer patients, where the FATHMM-MKL Score predicted the mutation to be not functionally significant.

Furthermore, we evaluated specific SNV and indel hotspots that came up as potential candidates in at least one of our analyses. For all these hotspots the evidence is sparse or suggests that they were caused by either other rearrangements in the genome, a specific mutational mechanism, or technical artifacts. These hotspots are described in detail in the Supplementary Note.

Even though we find an enrichment of highly mutated hotspots in multiple genomic regions and an enrichment of hotspots affecting known cancer genes, we have limited power to establish that individual hotspots are under positive selection. We provide a full list of hotspots in the PCAWG dataset (Supplementary Data 1–3), which includes multiple genomic features, measures for both CAF and expression, and prioritization of hotspots based on a combination of CAF and expression *z*-score evaluations.

## Discussion

Here we identified more than 700,000 site-specific hotspots, which is more than expected by chance under the given models for the background mutation rate. Nevertheless, various mutational processes not fully incorporated in existing models likely create most of these hotspots. Overall, the protein-coding genes were enriched for hotspots compared to the other regions, and the majority of protein-coding SNV hotspots were known cancer driver hotspots. Most protein-coding indel hotspots were associated with tumor suppressors, consistent with their ability to introduce frameshift and disrupt the protein product. We found that gene regulatory hotspots had an excess of potential same-patient second-hit missense mutations compared to singletons, but most of these were likely caused by mutational processes or artifacts. Based on our general characterization, we catalog hotspots and statistically evaluate their driver evidence.

The few known non-coding cancer drivers are related to genes that were already described as cancer genes. Both the two well-known *TERT* promoter hotspots^4,5^ and the newly identified hotspot in the 5’ UTR of *TP53*^13^, are in this catalog of hotspots. It is plausible that cancer genes may be affected through non-coding deregulation. Therefore, we hypothesize that non-coding hotspots associated with cancer genes a priori had a higher chance of being cancer drivers than non-coding hotspots associated with other genes. We observed enrichment in the proportion of cancer genes relative to non-cancer genes among protein-coding SNV and indel hotspots, and SNV hotspots in splice-sites, promoters, and enhancers. The enrichments among hotspots in the protein-coding genes as well as in the splice-sites and promoters can be explained by known driver hotspots^4,5,36,42^, whereas the enrichment among enhancer hotspots partly was caused by an SNV hotspot in an enhancer for *POU2AF1* that may be a non-coding cancer driver. The sparsity of non-coding driver candidates is however not surprising as recent studies exploring the non-coding genome find that non-coding drivers are present but less frequent than protein-coding drivers^13^.

In the protein-coding region of oncogenes, we only observe missense hotspot mutations, and we see an overall upward shift in expression compared to wild-types and singletons. This is as expected as missense mutations potentially activate the oncogenes at the protein level, and oncogene expression levels are often upregulated in cancer^43,44^.

We observe both missense and nonsense mutations, and frameshift and in-frame indels in the protein-coding region of tumor suppressors. The overall expression aberrations vary between mutation types. We see an overall lowered expression level for tumor suppressors with nonsense mutations and frameshift indels. In contrast, tumor suppressors with missense mutations have an elevated expression level compared to wild-types. The lowered expression level for nonsense mutations and frameshift indels is expected as these types of mutations lead to nonsense-mediated decay of transcripts. There are multiple plausible explanations for the elevated expression level of tumor suppressors with missense mutations. There might be a selective advantage of these missense mutations, which will suggest a functional impact of the specific amino-acid changes. Another explanation may be altered feedback mechanisms. Either the missense mutation causes a loss-of-function of the resulting protein and inhibits negative feedback, or transcripts with a missense mutation cause a rise in the overall transcription of the locus. Both mechanisms would lead to elevated expression. Singleton mutations are more likely to be neutral than hotspots. Therefore, if the presence of hotspots and elevated expression was caused by selection, we would expect the expression level of tumor suppressors with missense singletons to resemble wild-types. Opposite, feedback mechanisms would affect hotspots as well as singletons. If the tumor suppressor hotspot leads to a partial loss of tumor suppressor function and cancer progression triggers a natural upregulation of the tumor suppressor, the mutation would mitigate the negative impact. This scenario also explains why particular hotspots are under positive selection, as they only impact tumor suppressor functions that hinder tumor expansion, while other functions of the same gene that are beneficial for the cancer cells may be maintained. We do see an elevated expression level among tumor suppressors with missense singletons, which indicates that feedback mechanisms are the most likely cause of these expression aberrations.

To draw final conclusions on whether selection affects individual hotspots, we would need larger datasets and ultimately perturbation experiments focusing on one mutational hotspot at a time. Larger datasets of individual cancer types would provide power for the identification of cancer type-specific hotspots that potentially could be of clinical relevance and help guide personalized treatment. The overall evidence that many of the gene regulatory hotspots are cancer drivers was limited, but for a few hotspots, individual analyses suggest that they could be under positive selection. For several region types, we saw an excess of SNV hotspots associated with cancer genes that were caused by already known driver hotspots. For indel hotspots, we only saw an excess of cancer genes in protein-coding regions. Interestingly we also saw an enrichment of cancer genes among SNV enhancer hotspots, which was partly caused by a potential driver hotspot in an enhancer for *POU2AF1*. Amplification of *POU2AF1* has previously been shown to promote cancer development in multiple myeloma^45^, and a germline variant in the 3’ UTR of *POU2AF1* has recently been associated with susceptibility to lymphoma^46^. Furthermore, Chapuy et al.^47^ identify a super-enhancer for *POU2AF1* covering the potential enhancer hotspot identified in this current study. Multiple lines of evidence supported that this hotspot may be under positive selection, including its potential to create a TFBS for NF-E2-like transcription factors.

## Methods

### Dataset

For this study, 2279 whole cancer genomes from the Pan-Cancer Analysis of Whole Genomes (PCAWG) project under the International Cancer Genome Consortium^14^ were used. These genomes are a subset of the 2583 whitelisted PCAWG samples^14^, excluding melanomas and lymphoid malignancies. Furthermore, the sex chromosomes were excluded from this analysis. An initial analysis of our identified single nucleotide variant (SNV) hotspots on the full PCAWG dataset gave a reason for these exclusions.

In brief, both the melanomas and lymphoid malignancies had many cancer-specific hotspots caused by the heterogeneous mutational processes that are unique for these cancer types: UV induced mutations and activation-induced cytidine deaminase (AID) mutations, respectively. These hotspots would dominate the overall set and could possibly dilute potential driver signals. The sex chromosomes were excluded for technical reasons because they harbored more artifactual mutation calls than the autosomes.

### Detection of site-specific hotspots

We defined site-specific hotspots as genomic positions (for SNVs and insertions) or regions (for deletions) where two or more samples had a mutation of the given type. For the SNV and insertion hotspots, the mutation had to be in the exact same location across the patients. For SNVs the nucleotide change was allowed to vary between the patients, and for insertions the insert and insert size were allowed to vary. For the deletion hotspots, deletions could contribute to hotspots, if they were either fully or partially overlapping, or if the deleted nucleotides were right next to each other. Hotspot detection was done in R using the basic R package.

### Filtering of hotspots

We use different filters on our SNV and indel hotspots. The filters on indels also apply to singleton mutations. The filters used were the removal of duplicated indels, removal of indels in homopolymer runs, and a site-specific noise filter.

We found few duplicated insertions or deletions where different mutational callers had minor differences for the indel. This was either a shift in the start or end position for deletion, a difference in insert for insertion, or a disagreement on the number of reads covering the reference and alternative alleles. Both copies of the duplicates were removed. This affected 5499 out of 156,164 indel hotspots.

We here define that a hotspot is located on the border of a homopolymer run if it overlaps a stretch of identical nucleotides that are at least nine bp long. Indels located at the border of homopolymer runs are likely caused by replication slippage^33^, and therefore we remove these hotspots. This is not the case for SNVs so we annotated the SNV hotspots with whether or not they were located on the border of homopolymer runs given that they potentially can be miscalled indels when reads are not fully anchored on both sides of the homopolymer run.

We applied a site-specific noise filter to the 1000 SNV hotspots with most mutations across patients. This filter uses all normal control samples from the dataset to assess the position-specific noise level. For each non-reference nucleotide, *a* and each cohort $c \in C,$ the relative frequency of normal samples with at least two reads supporting *a* was calculated as $p\left( {a,c} \right) \in \left[ {0;100} \right].$ The noise-score was calculated using Eq. (1).

1 $${\mathrm{noise}} - {\mathrm{score}} = \mathop {\sum}\limits_{c \in C} {{\mathrm{log}}{}_{10}\left( {p\left( {a,\,c} \right) + 1} \right)}$$

We removed the hotspots with a noise score above 20, which was regarded as the hotspots with a high level of noise across the normal controls.

### Null models

We used two models to estimate the number of positions with a given number of mutations across patients: a simple binomial null model and a position-specific and sample-specific null model that accounts for various genomic features.

The binomial null model assumes a uniform distribution of mutations along the genome in every patient. Thus, the assumption is that the mutation rate is the same across patients and genomic regions. Using this model, we calculated the expected number of positions without mutations, with only one mutation across patients and with multiple mutations across patients (two to ten). This model was used to predict expectancies for SNVs, insertions, and deletions.

For SNVs and insertions, we used the basic R package to calculate the genome-wide expectations, by sampling from a binomial distribution. For deletions, we used the R package IRanges to calculate the genome-wide expected hotspot counts, by randomly shuffling the observed number and size of deletions within the chromosomes. We used median counts of 10,000 random shufflings. We use a basic binomial test to calculate *p*-values for the SNV and insertion null-models, and for the deletions, we calculate the *p*-values as the proportion of expected hotspots as extreme or more extreme as the observed. Correction for multiple testing was performed (see below).

The position-specific and sample-specific null model calculates the mutation probability for each position in the genome individually while taking cancer-specific and sample-specific elements into account. The details of the null model are described by Bertl et al.^31^. These probabilities were combined with scores for each mutation to predict the number of site-specific SNV hotspots with a given number of patients using a convolution-based approach as described by Juul et al.^32^. We used ncdDetect^32^ to calculate the expectations and *p*-values. Correction for multiple testing was performed (see below).

### Overlap with functional elements and internal hierarchy

The hotspots were overlapped with different genomic functional elements and were only allowed to overlap one element type. The functional elements included were protein-coding genes, protein-coding splice-sites, 5’ UTRs, 3’ UTRs, protein-coding promoters, non-coding splice-sites (located in UTRs or promoters), and enhancers. Their overlap was prioritized in the given order. Protein-coding splice-sites and non-coding splice-sites were then grouped. Hotspots located in the functional elements for protein-coding genes or in the coding region were assigned to that specific gene. The hotspots that overlapped neither of these regions were included as intronic/intergenic hotspots.

The hotspots in enhancers and intronic/intergenic regions were assigned to the nearest gene, based on the distance to the transcription start site (TSS). For some hotspots located in introns of genes with long intronic regions, this means that the hotspot may be assigned to another gene because the TSS of this other gene is closer to the mutated position.

Overlaps were determined using the overlapSelect function from Kent Source Tree Utilities using bed-files containing region information. Annotation of the nearest gene / TSS for enhancers and intronic/intergenic regions was done using the closest function from Bedtools. We looked for the nearest TSS in both directions using the parameters -s and -S in each run, and the distance was extracted using option-D.

### Stratification of hotspots

We used a set of 699 cancer gene census genes (v83)^38^ to stratify the groups of hotspots further into whether they were expected to affect cancer genes or non-cancer genes. The cancer genes were further divided into sets of oncogenes, tumor suppressor genes, and other cancer genes.

We used a set of protein-coding hotspots in known driver positions as a proxy for positive selection. For this, we used a list of known somatic cancer driver positions from the Cancer Genome Interpreter repository (https://www.cancergenomeinterpreter.org/mutations). This list was made as a combination of data from DoCM, ClinVar, and OncoKB as well as mutations found in the literature, which was manually validated^48^.

### Grouping of hotspots

SNV hotspots and indel hotspots were grouped individually but using the same grouping criteria.

The hotspots were grouped based on the functional element they overlapped. Then they were divided into groups based on the number of mutations in a hotspot. For some analyses the hotspot groups were merged into groups with four or more mutations for SNV hotspots and two or more mutations for indel hotspots, which can be regarded as cumulative hotspot groups.

Within all these groups, the hotspots were further divided based on the associated gene being either oncogene, tumor suppressor, other cancer genes, or non-cancer gene. For some analyses, all cancer genes were grouped as one group. We use a set of 699 curated Cancer Gene Census genes (CGC; v83) maintained by COSMIC^38^ to define oncogenes, tumor suppressors, and other cancer genes.

### Region-wise hotspot enrichments

For SNV hotspots with four or more mutations, SNV singletons, indel hotspots with two or more mutations, and indel singletons we calculated the enrichment or depletion of hotspots/singletons in a given region relative to hotspots/singletons in the intronic/intergenic region, where we would expect limited positive selection. We use a one-sided binomial test to calculate *p*-values using the mutation rate in the intronic/intergenic region as probability and “greater” as an alternative hypothesis in the binom.test function from basic R. Correction for multiple testing was performed (see below).

### Gene regulatory hotspots with corresponding missense mutations

To evaluate if an excess of the gene regulatory hotspots had missense mutations in the coding region of the same gene in the same patients we did a two-sided Fisher’s exact test. We compared the proportion of gene regulatory SNV (*n* ≥ 4) and indel (*n* ≥ 2) hotspots that had missense mutations in the same gene in at least one of the patients with the SNV and indel singletons that had missense mutations in the same gene in the same patient.

### Cancer gene enrichment analysis

To compare group hotspots in different functional classes, we calculated (i) the proportion of CGC genes in each cumulative group, (ii) the proportion of CGC genes among singleton mutations for each functional class, and (iii) the overall proportion of CGC genes among all protein-coding genes. We used these measures to calculate the fold-change (FC) enrichment of CGC genes in each cumulative group using Eqs. (2) and (3).

2 $${\mathrm{FC}}_{{\mathrm{overall}}} = \frac{{{\mathrm{Proportion}}\,{\mathrm{of}}\,{\mathrm{CGC}}\,{\mathrm{genes}}\,{\mathrm{in}}\,{\mathrm{cumulative}}\,{\mathrm{group}}}}{{{\mathrm{Proportion}}\,{\mathrm{of}}\,{\mathrm{CGC}}\,{\mathrm{genes}}\,{\mathrm{among}}\,{\mathrm{all}}\,{\mathrm{genes}}}}$$

3 $${\mathrm{FC}}_{{\mathrm{singletons}}} = \frac{{{\mathrm{Proportion}}\,{\mathrm{of}}\,{\mathrm{CGC}}\,{\mathrm{genes}}\,{\mathrm{in}}\,{\mathrm{cumulative}}\,{\mathrm{group}}}}{{{\mathrm{Proportion}}\,{\mathrm{of}}\,{\mathrm{CGC}}\,{\mathrm{genes}}\,{\mathrm{among}}\,{\mathrm{singleton}}\,{\mathrm{mutations}}}}$$

As the proportion of cancer genes in each cumulative group is a binomial distribution, we use Clopper-Pearson intervals as an approximation for the 95% confidence interval for the proportion of cancer genes in these groups. As the FC is a ratio of ratios, the confidence interval needs to be on the same scale. For the comparison with CGC genes among all genes, the upper and lower confidence interval boundaries are divided by the proportion of CGC genes among all genes or the proportion of CGC genes which is a constant (699/20,805). The proportion of CGC genes among singletons is not constant and varies between regions and mutation types, so they should be treated as binomially distributed ratios. As the amount of data for singletons is much higher than for hotspots (1700–8700x for SNVs; 25-41x for indels) we assume that the variance among singletons is so much lower than the variance among hotspots, and as these confidence intervals are approximations only used for visualization, we simply divide the confidence interval boundaries by the proportion of CGC genes among singletons. We use the binom.test function from basic R to calculate confidence intervals. We use a binomial test to calculate *p*-values with either the overall proportion of CGC genes among all genes or the proportion of CGC genes among singletons as a probability in the binom.test function in R. Correction for multiple testing was performed (see below).

### Evaluation of transcription factor binding peaks

For each protein-coding or gene regulatory hotspot with an ENCODE transcription factor binding peak score above 20, we evaluated the potential effect on transcription factor binding. Using the TomTom^49^ tool (v.5.1.1) from the MEME-Suite we searched the HOCOMOCO database (v.11 CORE) for transcription factor binding motifs using +/−5 bp around an SNV or indel, including the sequence with and without the mutation. For the search we used the following parameters: comparison function: Pearson correlation coefficient; significance threshold: *q*-value < 0.05; complete scoring activated; scoring of reverse complements activated. The tool uses a uniform background distribution of nucleotides, which is not entirely true, as A/T nucleotides take up around 60% of the human genome. The matches were manually checked using the logo-plots in the HTML output from TomTom to ensure that the hotspot position was important in the motif.

### Cancer allele fractions

The advantageous driver mutations are expected to be early events in cancer development, and therefore we expect them to have a high cancer allele fraction (CAF). The CAF is a variation of the variant allele fraction (VAF) corrected for tumor purity and copy number in the exact position. We calculate CAF using Eq. (4).

4 $${\mathrm{CAF}} = {\mathrm{VAF}} \cdot \frac{{{\mathrm{CN}} \cdot {\mathrm{TP}} + 2 \cdot \left( {1 - {\mathrm{TP}}} \right)}}{{{\mathrm{CN}} \cdot {\mathrm{TP}}}},$$

where ${\mathrm{CN}}$ is the copy number in the position and ${\mathrm{TP}}$ is the tumor purity of the patient sample.

High CAFs could also be caused by loss of heterozygosity, which will affect a larger region of the DNA. Therefore, we use the change in CAF between the mutation in the hotspot in the nearest mutation in the same patient, which should be at least 2 kilobases away from the hotspot and should be located outside protein-coding genes. This should give a robust measure that does not give high values if large regions of DNA are lost. We call this measure ΔCAF and calculate it using Eq. (5).

5 $$\Delta {\mathrm{CAF}} = {\mathrm{CAF}}_{{\mathrm{hotspot}}\,{\mathrm{mutation}}} - {\mathrm{CAF}}_{{\mathrm{nearest}}\,{\mathrm{mutation}}}$$

The ΔCAF values were *z*-score normalized using Eq. (6).

6 $${\mathrm{CAF}}\,z - {\mathrm{score}} = \frac{{\Delta {\mathrm{CAF}} - {\mathrm{mean}}(\Delta {\mathrm{CAF}})}}{{{\mathrm{std}}(\Delta {\mathrm{CAF}})}}$$

### Permutation tests of cancer allele fractions for protein-coding mutations

We used permutations to test the significance of the median ΔCAF *z*-score for the protein-coding hotspots and singletons. We performed 100,000 permutations. We shuffled labels of the ΔCAF *z*-scores, so each value was randomly assigned to either (i) known driver hotspots, (ii) hotspots in protein-coding regions of CGC genes excluding the known driver hotspots, (iii) hotspots in protein-coding regions of non-CGC genes, (iv) singletons in protein-coding regions of CGC genes excluding known driver positions, and (v) singletons in protein-coding regions of non-CGC genes. We downsampled singletons to include 10% of the singletons (*n* = 25,422) to speed up the permutations. All protein-coding hotspots were included (*n* = 9988). The median *z*-score was calculated for each group for each round in the permutation and was used as a null distribution within each group. As an approximation of the *p*-value we used the proportion of values above the true median *z*-score. Correction for multiple testing was performed (see below).

### Expression correlations

We had expression data for 979 of the 2179 patients included in this study. As the hotspots were identified pan-cancer, we used *z*-score normalized expression values to allow for comparison of values within hotspots with mutations across cohorts. The normalization was calculated using Eq. (7).

7 $${\mathrm{expression}}\,z - {\mathrm{score}} = \frac{{{\mathrm{expression}}\,{\mathrm{value}} - {\mathrm{mean}}({\mathrm{cohort}})}}{{{\mathrm{std}}({\mathrm{cohort}})}}$$

### Wild-type expression

To evaluate the expression levels, we used wild-type expressions in the same gene(s). For each specific gene, all patients that neither had copy number changes (copy number less than or greater than two) in the gene nor had SNVs or indels in the protein-coding region or regulatory regions (including splice-sites, promoters, and UTRs) of the gene, were included in the wild-type set for that gene.

### Wilcoxon rank-sum test of expression among groups of hotspots

We performed Wilcoxon rank-sum tests for hotspots stratified into 12 groups by region (protein-coding, splice-site, 5’ UTR, 3’UTR, promoter, and enhancer) and mutation type (SNV or indel). Within each group, hotspots were further divided into oncogenes, tumor suppressors, unclassified cancer genes, and non-cancer genes, as the expected effect on expression differed between these groups.

Calculations were done using the wilcox.test method from basic R, with hotspot expression z-scores as one group and corresponding wild-type expression z-scores as the other group. We performed a one-sided test for oncogenes (alternative hypothesis is greater) and tumor suppressors (alternative hypothesis is less), and a two-sided test for unclassified cancer genes and non-cancer genes. Correction for multiple testing was performed (see below).

### Ranking of hotspots using *p*-values

For each hotspot, we calculated a *p*-value for the ΔCAF *z*-score and the expression *z*-scores. These *p*-values were then combined using Fisher’s method^50^. The combined *p*-values are not used for evaluation of significance, rather we use the $- \log _2\left( {{\mathrm{p}} - {\mathrm{value}}} \right)$ as a way to rank hotspots.

The *p*-values for the ΔCAF *z*-score are calculated using a Wilcoxon rank-sum test where the ΔCAF *z*-scores for the hotspot is compared to ΔCAF *z*-score for singleton mutations in the same region for the same gene (e.g., singletons in the promoter of *TERT* are used for the *TERT* promoter hotspots). Here we use a one-sided test, where the null hypothesis is that the hotspot ΔCAF *z*-scores are greater than the singleton ΔCAF *z*-scores.

The *p*-values for the expression *z*-score are also calculated using a Wilcoxon rank-sum test where the expression *z*-scores for the hotspot is compared to the expression *z*-scores for the patients with wild-type expression in the same gene. Here we use a one-sided or two-sided test, depending on cancer gene type (see above).

For hotspots where we had insufficient data (i.e., missing expression data; no singleton mutations in same region/gene combination; missing ΔCAF values) to calculate either the ΔCAF or expression *p*-value, the other *p*-value was used for ranking. Hotspots missing both ΔCAF and expression *p*-values are ranked below the other hotspots and only based on the number of mutations in a hotspot.

Wilcoxon rank-sum tests were done using wilcox.test from basic R, while the combination of *p*-values was done with sumlogs from the metap R package.

### Multiple testing corrections

We performed multiple testing corrections for the *p*-values from the enrichment analyses, the permutation test of CAF for protein-coding mutations, and the *p*-values from the wilcoxon rank-sum test of expression among groups of hotspots. All corrections were done using the approach described by Benjamini and Hochberg^51^. The corrections result in *q*-values, where we consider findings significant if the *q*-value is below a chosen threshold for the false discovery rate. We used a threshold for the false discovery rate of 10% (*q*-values below 0.1) for the enrichment analyses, and 1% (*q*-values below 0.01) for both the CAF and expression analyses.

As the *p*-values for the individual hotspots (from Fisher’s method) only are used for ranking, they are not corrected for multiple testing.

### Mutational signature contributions

To evaluate the possibility that specific SNV hotspots were caused by various mutational processes we used a set of mutational signatures extracted using the full version of the dataset we use in this study. The identification of these signatures is described in detail by Alexandrov et al.^52^. We used the set of COMPOSITE signatures extracted using the SignatureAnalyzer method.

The probability of each signature was attributed to each mutation in a given patient based on the trinucleotide context around the mutation and the specific nucleotide exchange that happens in the given position in that specific patient.

For visualization, some signatures were summarized if they had the same proposed etiology in the paper by Alexandrov et al.^52^. This led to the following groupings:

APOBEC signatures: COMPOSITE signatures 2, 13, and 69.

Mismatch repair signatures: COMPOSITE signatures 6, 14, 15, 21, 26, 44, 73, 76, and 79.

UV signatures: COMPOSITE signatures 7a, 7b, 7c, 38, 55, 65, and 67.

POLE signatures: COMPOSITE signatures 9, 10a, 61, 62, 63, 66, and 78.

Treatment signatures: COMPOSITE signatures 11, 22, and 35.

Signature 17: COMPOSITE signatures 17a and 17b.

Prostate-specific signatures: COMPOSITE signatures 37 and 80.

Indel driven signatures: COMPOSITE signatures 64, 71, 77, 81, and 82.

Unknown signatures: COMPOSITE signatures 8, 12, 19, 28, 30, 33, 39, 68, 70, and 83.

Even though COMPOSITE signature 39 has unknown etiology it was not included in the unknown signatures as we go into detail with this signature.

For the hotspots, we calculated one value for each signature by taking the mean across the patients with mutations at the hotspot while taking differences in nucleotide exchange into account. When grouping hotspots, we also calculated one value for each signature by taking the mean across the hotspots.

### Genomic annotations

Each hotspot was annotated with various genomic features, which were used to evaluate their potential as cancer drivers. These annotations include the before-mentioned overlap with functional elements or nearest gene is located in an enhancer region, an intron or the intergenic region. This overlap dictates what gene a specific hotspot is associated with, and each hotspot was annotated with the CGC-status of the gene. As mentioned, the indel hotspots located in homopolymer runs were excluded, for the SNV hotspots, this feature was used as a genomic annotation. Further annotations include location in repeat masked elements, in genomic self-chains, in DNA level palindromes, in optimal APOBEC3A binding sites, if the position is known from dbSNP, various ENCODE chip-seq scores, and phast element conservation scores.

The list of repeat masked elements include short and long interspersed nuclear elements (SINEs; LINEs), long terminal repeats (LTRs), DNA and RNA repeats, low complexity repeat, satellite repeats, and simple repeats, which include micro-satellites. The list also includes repeats categorized as “other” and “unknown”. We downloaded this list of repeat masked elements from the Table Browser on the UCSC Genome Browser webpage (https://genome.ucsc.edu/). The table rmsk was downloaded for the whole genome and the columns genoName, genoStart and genoEnd described the location of the elements while the column repClass was used to classify the kind of repeat.

Human duplicated regions (self-chains) are regions where the human genome aligns with other regions in the human genome (excluding regions where a location maps to itself). The alignment algorithm allows for long gaps in both sequences. The outcome locates areas where the human genome has been duplicated. Mutations located in self-chain regions may be artificial since reads potentially map to multiple locations in the genome. The list of self-chain regions was also downloaded from the Table Browser on the UCSC Genome Browser webpage. The table chainSelf was chosen and the columns tName, Start, and tEnd described the location of the regions. Since these regions also include unaligned regions between stretches of aligned base pairs, candidate positions that were annotated as being located in self-chains were visually investigated in the UCSC Genome Browser.

DNA level palindromes are defined as in Rheinbay et al.^13^. We only annotate SNV hotspots located in the loop region of DNA level palindromes. This annotation is used to further support hotspots with high posterior probability for the APOBEC mutational process. Loop regions of palindromes sometimes overlap with homopolymer runs. In these cases, the palindrome loop annotation is considered an artifact caused by the homopolymer run, rather than support for the APOBEC mechanism.

We downloaded a list of the 706,999 most optimal binding sites for the APOBEC3A enzyme from Buisson et al.^53^ and overlapped all SNV hotspots with these sites. Together with the DNA level palindromes, this annotation was used as support for hotspots being caused by the APOBEC mechanism rather than selection.

We downloaded a list of common variants from dbSNP (build 151) using the Table Browser on the UCSC Genome Browser webpage. The common SNPs are SNPs with a minor allele frequency above or equal to 1% and they are restricted to the only map at one location in the genome. We used the table snp151Common, where the columns chrom, chromStart, chromEnd identified the positions of the common SNPs, and the rs-numbering was found in the column “name”. The type of mutation was found in the column “class”, where we used “single” (SNPs), insertion, deletion, and in-del.

We annotated each hotspot with various chip-seq scores for ENCODE-defined functional elements including transcription factor binding peaks, enhancer elements, DNase hypersensitive elements, and a variety of RNA elements.

We downloaded the phast element conservation scores using the Table Browser on the UCSC Genome Browser webpage. We used the table phastConsElements100way, where the column “score” holds the scores. We used the columns chrom, chromStart, and chromEnd to define regions with specific scores. Hotspots located in a given region get the corresponding score. Phast element conservation scores are described in detail by Siepel et al.^54^.

### Reporting summary

Further information on research design is available in the Nature Research Reporting Summary linked to this article.

## Supplementary information

Supplementery Information

Supplementary Data 1

Supplementary Data 2

Supplementary Data 3

Reporting Summary

## Data Availability

The dataset used for this study can be downloaded from the ICGC Data Portal under the PCAWG release: https://dcc.icgc.org/releases/PCAWG. Additional information on accessing the data can be found at https://docs.icgc.org/pcawg/data/. Most of the data is publicly available, but all the TCGA data and the potentially identifiable data from ICGC are protected and need permission to download. To access the protected data, researchers will need to apply to the TCGA Data Access Committee (DAC) via dbGaP (https://dbgap.ncbi.nlm.nih.gov/aa/wga.cgi?page=login), and the ICGC Data Access Compliance Office (DACO; http://icgc.org/daco), respectively.

## Code availability

All analyses in this study were performed using R version 3.5.1, bedtools version 2.29.2 or ucsc-overlapselect version 366. Besides using basic R, we used the R packages IRanges, dplyr, and metap. Custom R scripts used for the individual analyses are available upon request.
