# Supplementary material for: The landscape and driver potential of site-specific hotspots across cancer genomes
Source: NPJ Genom Med. 2021 May 13;6:33. doi: 10.1038/s41525-021-00197-6 (PMC8119706; doi:10.1038/s41525-021-00197-6)
Supplement: Supplementary file 1 — Supplementery Information [file 41525_2021_197_MOESM1_ESM.pdf]

Supplementary Information for "The landscape and driver potential of site-specific hotspots across cancer genomes"

Randi Istrup Juul[1*], Morten Muhlig Nielsen[1], Malene Juul[1], Lars Feuerbach[2], Jakob Skou Pedersen[1,3*]

[1] Department of Molecular Medicine, Aarhus University Hospital, Denmark
[2] Division of Applied Bioinformatics, German Cancer Research Center, Im Neuenheimer Feld 280, Heidelberg 69120, Germany
[3] Bioinformatics Research Centre, Aarhus University, Denmark
[*] Corresponding authors

This supplementary information includes:

Supplementary Notes 2
Overview of Supplementary Figures, Tables and Data 11
Supplementary Figure 1 12
Supplementary Figure 2 13
Supplementary Figure 3 14
Supplementary Figure 4 15
Supplementary Figure 5 16
Supplementary Figure 6 17
Supplementary Figure 7 18
Supplementary Figure 8 19
Supplementary Figure 9 20
Supplementary Table 1 22
Supplementary Table 2 23

Supplementary Notes

1. Signature differences between known drivers and other hotspots

2. Detailed description of hotspots without strong signs of positive selection

1. Signature differences between known drivers and other hotspots

We summarized contributions of a set of mutational signatures (from Alexandrov *et al.*[1]) across all SNV hotspots to compare hotspots in various genomic regions (**Methods**). We compare signature contributions among all SNV hotspots ($n \geq 2$) in protein-coding regions. They are divided into hotspots in driver positions, in cancer genes and in other genes. We only find a few major differences between these groups. The biggest differences between driver hotspots and hotspots in cancer genes and other genes are the mean contribution of signatures related to altered activity of polymerase ε (POLE) or signature 39, for which the etiology is unknown.

The contribution of the POLE signatures are very low in the known driver hotspots compared to hotspots in cancer genes and other genes (**Supplementary Fig. 7**). It has recently been demonstrated that POLE exonuclease domain mutations give rise to driver mutations in a specific trinucleotide context in specific genes[2]. The set of known drivers here include many more contexts than these, which may explain the low contribution of the POLE signature among the known drivers.

The contribution of signature 39 varies between known driver hotspots, hotspots in cancer genes, and hotspots in other genes, with highest contribution in drivers and lowest in non-cancer genes (**Supplementary Fig. 7**). We found the same trend for both splice-site hotspots and promoter hotspots, where the hotspots in cancer genes had higher contribution of signature 39 than non-cancer gene hotspots. Neither the many *TP53* splice-site hotspots, nor the *TERT* promoter hotspots could fully explain the higher contributions of signature 39 among the cancer gene hotspots. Signature 39 may capture background mutational processes as it has an unknown etiology and it is a mixed signature with most C>G mutations in various contexts but also a fair amount of C>A, C>T, T>A, T>C and T>G mutations in almost all contexts. The mixed nature of signature 39 makes it plausible that it captures background mutational processes, however it does not explain why we observe a higher contribution of this signature among the known drivers. A recent study uses nucleotide context to detect cancer drivers, as they find an enrichment of passenger mutations in specific contexts, which depends on active mutational processes and tumor type. On the contrary, they say that drivers are localized in important functional positions, which are independent of nucleotide context, thereby leading to more drivers in unusual contexts[3]. The mixed mutational landscape of signature 39 may though result in mutations in so-called unusual contexts, which could explain why hotspots in known drivers have a higher contribution of this signature than other hotspots.

2. Detailed description of hotspots without strong signs of positive selection

Gene regulatory hotspots with multiple missense mutations in the corresponding protein-coding gene

We found three SNV and four indel gene regulatory hotspots with multiple patients that had missense mutations in the corresponding gene. None of them have convincing signs of positive selection.

SNV hotspots

The three SNV hotspots had high probabilities for the POLE mutational signatures. Altered activity of POLE can potentially cause hypermutability[4,5], which may increase the chance of having both protein-coding and gene regulatory mutations related to the same genes for these patients. These hotspots were located in an enhancer near *PELI2* (25 kilobases [kb] away) and in two 3' UTR for *TRIM36* and *TAX1BP1*. All the hotspots had four mutations across patients and have at least two patients with coding missense mutations in the same gene. The *PELI2* enhancer hotspots were only found in colorectal adenocarcinoma patients, while the two 3' UTR hotspots both had three mutations in colorectal adenocarcinoma patients and one in a uterus adenocarcinoma patient. Both cancer types are known to have subtypes with POLE mutations. The top-3 signatures vary between 10a, 61, 62, and 66, which all are POLE signatures, and the combined probability for the top-3 signatures were 92%, 77% and 91% for these hotspots. None of the hotspots were located in homopolymer runs, known from dbSNP, or located in palindromic regions, repeats or duplicated regions. We have ΔCAF data for all patients in these hotspots, and only the *TRIM36* hotspot had an above-median ΔCAF z-score. We have expression data for all patients in these hotspots as well, and only the *TAX1BP1* hotspot had large expression aberrations compared to wild-type expression. None of the hotspot are in conserved phast elements nor have high ENCODE scores for any ENCODE region.

Indel hotspot in enhancer of *EGFR*

We found an indel hotspot in an enhancer of the *EGFR* oncogene that has six insertions, of which four are in patients with glioblastoma. Mutations within *EGFR* and amplification of the gene or specific domains of the gene is well known in glioblastoma[6–9]. The remaining two mutations are in patients with uterus adenocarcinoma and bladder transitional cell carcinoma. Three of the six patients did also have protein-coding missense mutations in *EGFR.* Overall, we found seven missense mutations in *EGFR* across these patients. According to the Genomics of Drug Sensitivity in Cancer database (GDSC)[10], there exists 16 different anticancer drugs targeting *EGFR* mutated cancers. Our dataset holds expression data for five of the patients. Even though the expression values for these five patients were variable (z-scores from -0.3 to 2.96), we observed an overall upregulation of *EGFR* expression compared to wild-type expression of *EGFR* (**Supplementary Fig. 6**). In general, the region around this insertion seemed to be rather unproblematic given that the insertion did not overlap repeat masked elements or duplicated regions (**Fig. 4**). The specific insertion seemed promising since it was the same dinucleotide insert (GT) across all six patients, but a closer look at the surrounding DNA sequence revealed that this insertion are located at the border of a stretch of five repeated GTs, suggesting that this specific insertion may be a result of replication slippage, which causes either insertions or deletions of various repeats (from mono- to oligonucleotide repeats)[11]. Furthermore, the VAFs of this insertion were very

low for five of the patients (< 0.01). This was caused by extremely high coverage of this region (460-2100x) and very few reads supporting the mutation (5-11). This suggested that this region could be amplified in these patients, and copy number analysis revealed that this was indeed the case. The five patients with extremely low VAFs had copy numbers between 49 and 126 in this region with only a few copies of the minor allele. Moreover, we saw similar copy numbers in the coding region of *EGFR* in these patients. Thus, a more plausible explanation for the upregulation in gene expression is that the gene itself is amplified rather than the insertion in this specific enhancer hotspot.

Other indel hotspots

The remaining three indel hotspots all only had two mutations across patients. These were a 1 base pair (bp) deletion hotspot in the promoter of *CCDC88A* with mutations in two colorectal adenocarcinoma patients, a 1 bp deletion hotspot in the 3' UTR for *NAV3* with mutations in one colorectal adenocarcinoma patient and one stomach adenocarcinoma patient, and a 1 bp insertion hotspot in the 3' UTR for *BNC2* with mutations in two colorectal adenocarcinoma patients. None of these hotspots were known from dbSNP nor located in repeats or duplicated regions, and none of the hotspots have high ENCODE scores for any ENCODE region. We had expression data for all three hotspots, and all showed a small increase in expression compared to the wild-type expression levels. The *CCDC88A* promoter hotspot had a ΔCAF z-score near zero, whereas both 3' UTR hotspots had negative ΔCAF z-scores. The *NAV3* 3' UTR hotspot were located in a conserved element with a phast element score of 737.

High frequency hotspots

Non-cancer gene regulatory SNV hotspots

We found four SNV hotspots with more than ten mutations associated with non-cancer genes. Two of these were located in an intronic enhancer for *GPR126* with 19 and 12 mutations across patients, the other two were located in the *PLEKHS1* promoter and both had 16 mutations across patients. Both the two *GPR126* enhancer hotspots and the two *PLEKHS1* promoter hotspots were located very close to each other with only two nucleotides between them. All four hotspots were located in the loop region of a palindromic sequence, which is believed to form DNA-level hairpins that may be targets for APOBEC enzymes. The two hotspots in the *PLEKHS1* promoter have previously been described[12] and shown to have high contributions of the APOBEC mutational signatures[13]. Here we found that all four hotspots have high probabilities for the APOBEC signatures (signatures 2 and 13), with 90% and 33% for the *GPR126* enhancer hotspot and 93% and 25% for the *PLEKHS1* promoter hotspot. Furthermore, both the *GPR126* enhancer hotspot and the *PLEKHS1* promoter hotspot with APOBEC probabilities around 90% were located in optimal APOBEC3A binding sites.

None of these hotspots were located in homopolymer runs, known from dbSNP, located in repeats or duplicated regions, nor have high ENCODE scores for any ENCODE region. We have ΔCAF data for all patients in these hotspots, and the two *GPR126* enhancer hotspots and one of the *PLEKHS1* promoter hotspots had above-median ΔCAF z-scores. We have expression data for 9-15 patients in these hotspots, but none had large expression aberrations compared to wild-type expression. One of the *GPR126* enhancer hotspots are

located in a conserved element with a phast element score of 400. In summary, these four hotspots are likely driven by APOBEC editing[12,13].

Intronic / intergenic hotspots

We found 34 SNV and five indel hotspots in the intronic/intergenic region. Of these 34 SNV hotspots, only two were related to cancer genes. One was an intergenic hotspot with 14 mutations near the oncogene *BCL11A* and the other was an intronic hotspot with 11 mutations in a *KIAA1598* intron. The remaining 32 hotspots were related to the genes *SLITRK3, FCRLA, ZNF93, PRDM14, COL11A1, ANKRD30A, CCDC85A, TFAP2B, GYPE, TP53TG3, GSDMC, ANGPT1, ZNF737, FLJ00325, OSTF1, C12orf50, CHRM2, KCNQ5, HNF4G, YTHDF3, METTL15, HCN1, MYT1L, HGSNAT, CNTNAP5, NRXN1, AC006455.1, AC011239.1, BASP1, C7orf33, CDH9,* and *UQCRFS1*. The COSMIC database (v. 92)[14] had few mutations in 20 of these 32 hotspots (1-5 mutations per hotspot), and all but one of them had not functionally significant FATHMM-MKL Scores. The five intronic/intergenic indel hotspots were associated with the non-cancer genes *APOH, DKFZP547L112, ALPK2, NTM,* and *TMEM114*.

SNV hotspot in intergenic region near *BCL11A*

All 14 patients with mutations in the *BCL11A* hotspot had prostate adenocarcinoma. The VAFs for all 14 patients were very low. In the BAM-files and VCF-files we found that in general only a few reads supported the variant in these patients even though the read depth was normal. Furthermore, we found that the reads supporting the variant were problematic. For some patients the base quality of the variant was low and for other patients the variant was always observed together with two other variants 3 bp and 5 bp upstream the hotspot mutation. These mutations were not present in the corresponding normal samples nor did they pass the quality filters and were therefore not called as SNVs. This indicates that this hotspot is an artifact.

SNV hotspot in intron of *KIAA1598*

The hotspot in an intron for *KIAA1598* were located 38 kb from the transcription start site (TSS). Among the top-3 signatures for this hotspot was signatures 1, 30, and 8 (probability: 22%; 15%; 14%). Oddly, the hotspot is not located in a CpG site, which is almost always the case for mutations with high signature 1 probabilities. Instead it is found in a highly repetitive region with many triple adenines separated by a single thymine or cytosine. The ΔCAF z-score was very negative and we did not see expression aberrations when comparing with wild-type expression. This hotspot is not located in a homopolymer run, known from dbSNP, or located in a palindromic region, repeat or duplicated region. The hotspot is not in a conserved phast element nor have high ENCODE scores for any ENCODE region. To summarize, we find no clear explanation for this hotspot.

SNV hotspots associated with non-cancer genes

Of the 32 SNV hotspots associated with non-cancer genes two were located on the border of homopolymer runs, one of which were also in a loop of a palindromic sequence, i.e. it is located between two homopolymer runs of complementary nucleotides. Seven of the hotspots are located in duplicated regions, and 19 are located in repeats (7 LINE segments, 10 LTR segments, 2 SINE segments). In relation to sequencing and mutation calling, all

these regions are error-prone. None of them are known from dbSNP or located in palindromic sequences (except the before mentioned homopolymeric one). A single of these hotspots had a high ENCODE score for transcription factor binding peaks (TFP; 84). Among the 11 SNV hotspots that are located outside error-prone regions, we find an intergenic hotspot located 95 kb from the TSS of *GSDMC*. The hotspot is located in an ENCODE TFP, but neither the reference nor the alternative sequence around the hotspot match transcription factor binding motifs. The other ten hotspots had a majority of patients with either esophagus- or stomach adenocarcinoma (53-85%). Further, signatures 17a and 17b contributed the most to these (49-96% combined), which are signatures of unknown etiology that are enriched among esophagus- and stomach adenocarcinoma patients. This indicates that these hotspots are likely caused by a mutational mechanism. One of these ten hotspots is located in a conserved element with a phast element score of 381 (hotspot near *KCNQ5*), and this is also the hotspot with a functionally significant FATHMM-MKL Score in the COSMIC database. Besides the *GSDMC* hotspot, four others have high ENCODE scores (78-87) for TFPs (hotspots near *SLITRK3, OSTF1, C12orf50, and KCNQ5*). Two of these ten hotspots had an above-median ΔCAF z-score (hotspots near *OSTF1* and *FLJ00325*), and three had expression aberrations compared to the wild-type expression, but only few patients with expression (hotspots near *FLJ00325*, *HNF4G*, and *MYT1L*).

Indel hotspots

Two of the five indel hotspots were located in repeat regions of the DNA type and another one was located in a duplicated region. The two hotspots in non-error-prone regions are deletion hotspots near *APOH* and *ALPK2*. The *APOH* hotspot has 11 deletions and is located 19 kb from the TSS. It has an above-median ΔCAF z-score and only a small rise in expression. To summarize, this hotspot may happen early in cancer development, but it has no clear explanation. The *ALPK2* hotspot has 27 deletions and is located 159 kb from the TSS. It has an ENCODE TFP score of 15 and a small drop in expression. Furthermore, the *ALPK2* hotspot is located near *MIR122* and is potentially caused by transcription associated mutagenesis[15].

Hotspots with potential gain or loss of transcription factor binding site

We found three additional gene regulatory SNV hotspots and two protein-coding indel hotspots with a potential gain or loss of transcription factor binding site. The three SNV hotspots are located in a 5' UTR of *C1orf159*, a 3' UTR of *PI15* and a *NRD1* promoter, and the two indel hotspots are located in the protein-coding region for *VHL* and *RASAL2*.

SNV hotspot in 5' UTR of *C1orf159*

The hotspot in the 5' UTR of *C1orf159* had five mutations across patients, two with lung squamous cell carcinoma and the three patients with uterus adenocarcinoma, bladder transitional cell carcinoma and head and neck squamous cell carcinoma, respectively. The hotspot overlaps an ENCODE TFP with a score of 74, and the reference sequence around this hotspot, including the hotspot itself, match a E2F2 transcription factor binding site. Across the five patients three different nucleotide exchanges happen at the position, all interrupting the binding site, leading to a potential loss of transcription factor binding site. Even though the hotspot is not located in a palindromic region, two of the top-3 signatures are the APOBEC signatures, which have a combined probability of 70% for this hotspot.

Furthermore, the hotspot is located in an optimal APOBEC3A binding site, supporting it being caused by APOBEC editing. The discrepancy between the non-overlap with palindromic loop region and the optimal APOBEC3A binding site arise because of differences in the definition of palindromes in the datasources. Rheinbay *et al.*[15] requires a stem of at least 6 bp whereas Buisson *et al.*[16] only requires at least 3 bp. The ΔCAF z-score is between the 80th and 90th percentile for this hotspot indicating that it may have happened early in the cancer development. The hotspot is not known from dbSNP, not located on the border of a homopolymer run or in a conserved phast element, nor experience expression aberrations compared to wild-type expression. It is, however, located in a low-complexity type repeat region and a duplicated region.

SNV hotspot in 3' UTR of *PI15*
The hotspot in the 3' UTR of *PI15* had five mutations across patients, two with liver hepatocellular carcinoma and three with esophagus adenocarcinoma. The hotspot overlaps an ENCODE TFP with a score of 82, and the reference sequence around and including this hotspot, match CTCF and CTCFL transcription factor binding sites. Across the five patients three different nucleotide exchanges happen at the position, which all lead to a potential loss of the transcription factor binding sites. Furthermore, this hotspot has three mutations in the COSMIC database, where the FATHMM-KML Score predicts them to be pathogenic. The top-3 signatures include signature 12, 17b, and 28 with probabilities of 15-22%. The hotspot is located in a conserved element with a phast element score of 447. The ΔCAF z-score between the 70th and 80th percentile. The hotspot was not located in homopolymer runs, known from dbSNP, or located in palindromic regions, repeats or duplicated regions. We did not have expression data for this hotspot.

SNV hotspot in promoter of *NRD1*
The hotspot in the *NRD1* promoter had four mutations across patients, all in esophagus adenocarcinoma. The hotspot overlaps an ENCODE TFP with a score of 136, and in four of the patients the mutation in the hotspot results in the potential gain of a transcription factor binding site for E2F7 and FLI1. Signatures 17a and 17b are among the top-3 signatures for this hotspot with a combined probability of 81%, but as these signatures are highly prevalent among esophagus adenocarcinoma this is not surprising. We found a single mutation in this hotspot in an esophagus cancer in the COSMIC database, where the FATHMM-KML Score was not statistically significant. The hotspot was not located in homopolymer runs, known from dbSNP, or located in palindromic regions, repeats or duplicated regions, nor was it in a conserved phast element. The ΔCAF z-score was above-median, and furthermore, the z-scores for the singletons in this region are at the same level. We did not have expression data for this hotspot.

Indel hotspots
The hotspot in the protein-coding region of the tumor suppressor *VHL* has a one bp insertion in two patients with kidney renal cell carcinoma. This hotspot overlaps an ENCODE TFP with a score of 106, and the reference sequence without the insert matches a ZBTB6 transcription factor binding motif, which the insert potentially interrupts. The hotspot in the protein-coding region of *RASAL2* has a three bp deletion in two patients, one with prostate adenocarcinoma and one with biliary adenocarcinoma. This hotspot overlaps an ENCODE

TFP with a score of 231, and the alternative sequence including the deletion match a STAT6 transcription factor binding motif. As the reference sequence without the deletion does not match this motif, the hotspot mutation potentially leads to a gain of this binding site. Not surprisingly, as both hotspots are in protein-coding genes, they are located in conserved elements with phast element scores of 304 and 490. None of the hotspots were known from dbSNP, or located in palindromic regions, repeats or duplicated regions. Both hotspots had very negative ΔCAF z-scores. We only have expression data for one patient from each hotspot so no expression analyses were performed for these.

Cancer gene associated hotspots that have either above-median ΔCAF z-scores or expression aberrations

Besides the *POU2AF1* enhancer hotspot (included in the main text) and the *EGFR* enhancer hotspot (see section on gene regulatory hotspots with more than one missense mutation in the corresponding protein-coding gene), we also found an SNV hotspot in the *FGFR2* promoter and indel hotspots in the 5' UTR of *NUP214* and 3' UTRs of *CTNNA2* and *CCND1* that had either a high ΔCAF z-score or expression aberrations. We exclude SNV hotspots in the *TERT* promoter and *TP53* splice-sites, and the indel hotspots with only two mutations.

SNV hotspot in promoter of *FGFR2*

We found an SNV hotspot in the promoter of the oncogene *FGFR2*. This hotspot harbors mutations in six patients with cancers in five different tissues, including breast adenocarcinoma and lobular carcinoma, and bladder transitional cell carcinoma. A single mutation in this hotspot in a breast cancer patient is found in the COSMIC database with a not statistically significant FATHMM-KML Score. According to the GDSC database, three different anticancer drugs targeting *FGFR2* mutated cancers exist. We have expression data for five of these patients, and the median expression for these patients are downregulated compared to wild-type expression of *FGFR2* (**Supplementary Fig. 6**). The ΔCAF z-score was above-median. This hotspot is located in an ENCODE TFP with a score of 19. Furthermore, the analysis of mutational signature contribution to SNV hotspots suggests that this specific hotspot may be driven by APOBEC editing with a posterior probability of 68%. As APOBEC presumably acts on single-stranded DNA in DNA-level hairpins[13,16], the high probability for the APOBEC signatures is further supported by the hotspot being located in a loop of a palindromic region (**Fig. 4**), and by its location in an optimal APOBEC3A binding site. Moreover, the mutational mechanism of APOBEC editing has previously been found active in breast and bladder cancers[13,17,18]. Taken together these observations suggest that this hotspot may be caused by a mutational process rather than positive selection.

Indel hotspot in 5' UTR of *NUP214*

We found an indel hotspot in a 5' UTR of the cancer gene *NUP214* that has three deletions in three different cancers. All three mutations are a deletion of a single G in a non-repetitive context. We only have expression data for one patient, which shows a downregulation of the *NUP214* expression (-0.15). The ΔCAF z-score was around the 80th percentile (1.28) which suggests that the mutations may have happened early. Furthermore, this hotspot is located in an ENCODE TFP with a score of 118. In conclusion, ΔCAF z-score and the location in an ENCODE TFP indicate that this may be under positive selection, but the remaining evidence cannot verify or disprove this so further analysis will be needed.

Indel hotspot in 3’ UTR of *CTNNA2*

We found an indel hotspot in the 3’ UTR of the oncogene *CTNNA2*. It has four deletions across three cancer types, all being a deletion of a single nucleotide. We had expression data for two of the patients with this deletion, and the median expression level was slightly decreased compared to the median wild-type expression of *CTNNA2*. The individual expression z-scores were -0.30 and -0.49. The hotspot has a phast element conservation score of 543, indicating that this hotspot is located in a highly conserved element. The ΔCAF z-score was above-median, with a single individual negative z-score. The high phast element conservation score and the above-median ΔCAF z-scores may suggest that this position is under positive selection, while the negative ΔCAF z-score and the low expression values suggest otherwise.

Indel hotspot in 3’ UTR of *CCND1*

We found an indel hotspot in the 3’ UTR of the oncogene *CCND1* with three deletions. This hotspot was in a region with high sequence identity (**Fig. 4**). Visual inspection showed that it was located in a region between nearly identical stretches. The median expression level was elevated compared to the median wild-type expression of *CCND1*, but the values had high variance (**Supplementary Fig. 6**). The hotspot has a phast element conservation score of 455, which indicates that this hotspot is located in a highly conserved element. Two of the three deletions had unusual high read depths and very low support for the variant (7/285 and 8/841 reads support variant), therefore we did not evaluate the ΔCAF z-scores in this hotspot. We also found an SNV hotspot with three mutations in the 3’ UTR of this gene. In conclusion, the repetitive nature of the region and read-depth issues for two of three deletions suggests that this may be an artifact.

References

1. Alexandrov, L. B. *et al.* The repertoire of mutational signatures in human cancer. *Nature* **578**, 94–101 (2020).
2. Poulos, R. C., Wong, Y. T., Ryan, R., Pang, H. & Wong, J. W. H. Analysis of 7,815 cancer exomes reveals associations between mutational processes and somatic driver mutations. *PLoS Genet.* **14**, e1007779 (2018).
3. Dietlein, F. *et al.* Identification of cancer driver genes based on nucleotide context. *Nat. Genet.* **52**, 208–218 (2020).
4. Campbell, B. B. *et al.* Comprehensive Analysis of Hypermutation in Human Cancer. *Cell* **171**, 1042–1056.e10 (2017).
5. Ahn, S.-M. *et al.* The somatic POLE P286R mutation defines a unique subclass of colorectal cancer featuring hypermutation, representing a potential genomic biomarker for immunotherapy. *Oncotarget* vol. 7 68638–68649 (2016).
6. Frederick, L., Eley, G., Wang, X. Y. & James, C. D. Analysis of genomic rearrangements associated with EGRFvIII expression suggests involvement of Alu repeat elements. *Neuro. Oncol.* **2**, 159–163 (2000).
7. Biernat, W., Huang, H., Yokoo, H., Kleihues, P. & Ohgaki, H. Predominant expression of mutant EGFR (EGFRvIII) is rare in primary glioblastomas. *Brain Pathol.* **14**, 131–136 (2004).
8. Huang, H.-J. S. *et al.* The enhanced tumorigenic activity of a mutant epidermal growth factor receptor common in human cancers is mediated by threshold levels of constitutive tyrosine phosphorylation and unattenuated signaling. *J. Biol. Chem.* **272**, 2927–2935 (1997).
9. Wong, A. J. *et al.* Structural alterations of the epidermal growth factor receptor gene in human gliomas. *Proc. Natl. Acad. Sci. U. S. A.* **89**, 2965–2969 (1992).
10. Yang, W. *et al.* Genomics of Drug Sensitivity in Cancer (GDSC): a resource for therapeutic biomarker discovery in cancer cells. *Nucleic Acids Res.* **41**, D955–61 (2013).
11. Taylor, M. S., Ponting, C. P. & Copley, R. R. Occurrence and consequences of coding sequence insertions and deletions in Mammalian genomes. *Genome Res.* **14**, 555–566 (2004).
12. Weinhold, N., Jacobsen, A., Schultz, N., Sander, C. & Lee, W. Genome-wide analysis of noncoding regulatory mutations in cancer. *Nat. Genet.* **46**, 1160–1165 (2014).
13. Nik-Zainal, S. *et al.* Landscape of somatic mutations in 560 breast cancer whole-genome sequences. *Nature* **534**, 47 (2016).
14. Tate, J. G. *et al.* COSMIC: the Catalogue Of Somatic Mutations In Cancer. *Nucleic Acids Res.* **47**, D941–D947 (2019).
15. Rheinbay, E. *et al.* Analyses of non-coding somatic drivers in 2,658 cancer whole genomes. *Nature* **578**, 102–111 (2020).
16. Buisson, R. *et al.* Passenger hotspot mutations in cancer driven by APOBEC3A and mesoscale genomic features. *Science* **364**, (2019).
17. Lawrence, M. S. *et al.* Mutational heterogeneity in cancer and the search for new cancer-associated genes. *Nature* **499**, 214–218 (2013).
18. Roberts, S. A. *et al.* An APOBEC cytidine deaminase mutagenesis pattern is widespread in human cancers. *Nat. Genet.* **45**, 970–976 (2013).

Overview of Supplementary Figures, Tables and Data

Supplementary Figure 1:
Heatmap of hotspot enrichment / depletion across various genomic regions.

Supplementary Figure 2:
Distribution of SNV hotspots across regions.

Supplementary Figure 3:
Distribution of indel hotspots across regions.

Supplementary Figure 4:
Fold-change enrichment of hotspots in cancer genes.

Supplementary Figure 5:
Distribution of expression z-scores for patients with protein-coding indel hotspots.

Supplementary Figure 6:
Distribution of expression z-scores for patients with hotspot mutations.

Supplementary Figure 7:
Signature contributions among hotspots.

Supplementary Figure 8:
Distribution of expression z-scores for patients with SNV hotspots.

Supplementary Figure 9:
Distribution of expression z-scores for patients with indel hotspots.

Supplementary Table 1:
Significance evaluation of expectations under null-models (q-values)

Supplementary Table 2:
Significance evaluation of expression abbreviations (q-values)

Supplementary Data 1:
SNV hotspots with four or more mutations

Supplementary Data 2:
SNV hotspots with two or three mutations

Supplementary Data 3:
Indel hotspots with two or more mutations

Supplementary Figure 1

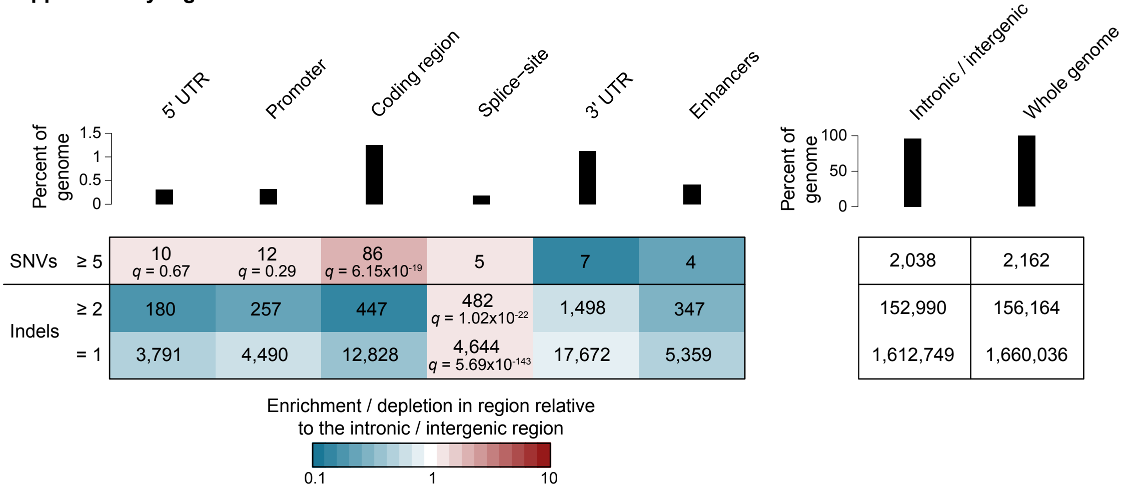

Supplementary Figure 1 | Heatmap of hotspot enrichment / depletion across various genomic regions. Bars show the genomic extent of each region; indels include indels in homopolymer runs; for the protein-coding and gene regulatory regions, $q = 1$ when not stated explicitly. UTR: Untranslated region.

Supplementary Figure 2

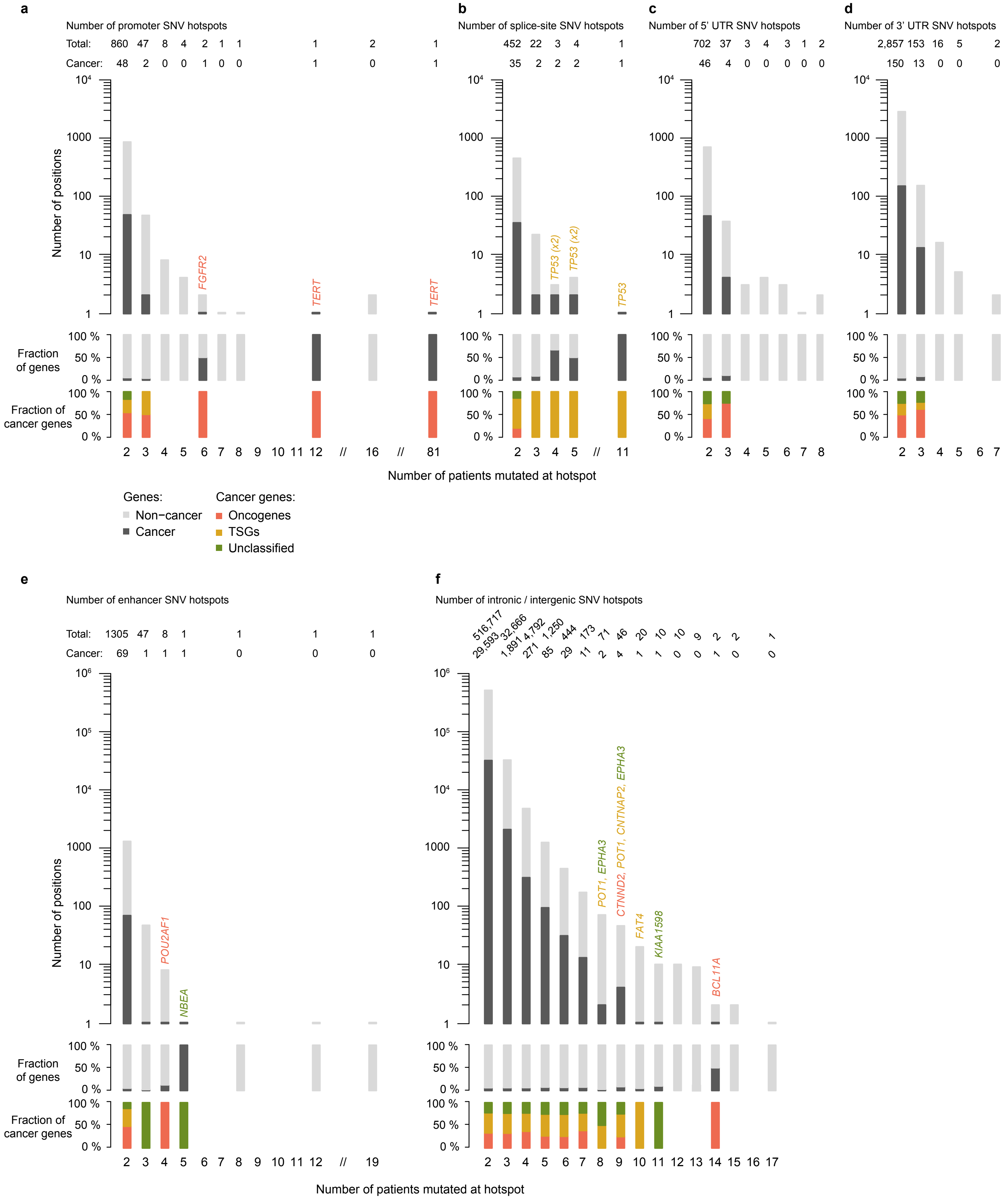

Supplementary Figure 2 | Distribution of SNV hotspots across regions. a-e. Bar-charts of promoter (a), splice-site (b), 5' UTR (c), 3' UTR (d), enhancer (e), and intronic / intergenic (f) hotspots; numbers above bars indicate the total number / number of cancer gene hotspots; the middle bar-plot show the fractions of cancer genes among the hotspots; the lower bar-plot show the fraction of oncogenes, tumor suppressors, and unclassified cancer genes in the total amount of cancer genes. TSG: Tumor suppressor gene; UTR: Untranslated region.

Supplementary Figure 3

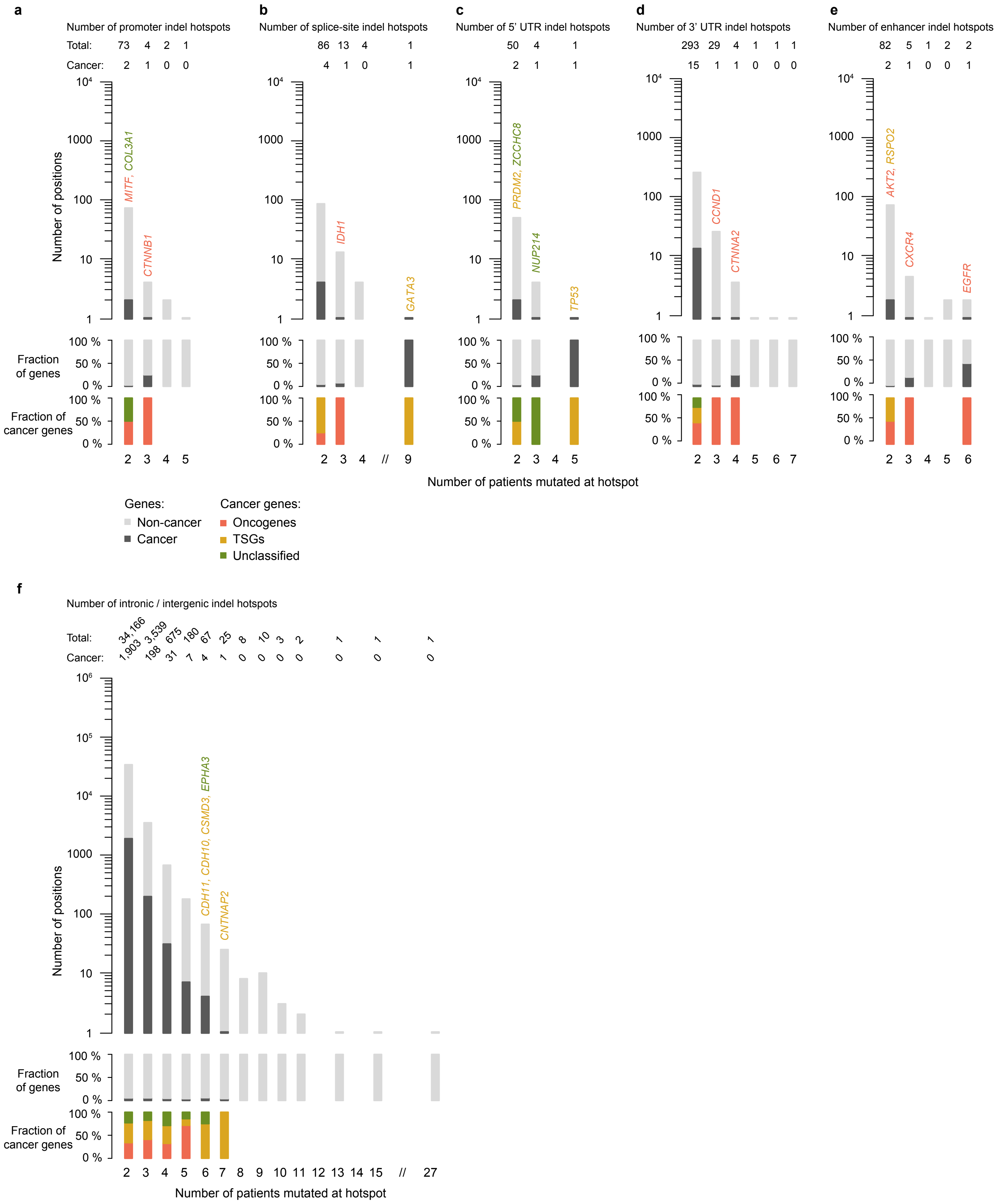

Supplementary Figure 3 | Distribution of indel hotspots across regions. a-e. Bar-charts of promoter (a), splice-site (b), 5' UTR (c), 3' UTR (d), enhancer (e), and intronic / intergenic (f) hotspots; numbers above bars indicate the total number / number of cancer gene hotspots; the middle bar-plot show the fractions of cancer genes among the hotspots; the lower bar-plot show the fraction of oncogenes, tumor suppressors, and unclassified cancer genes in the total amount of cancer genes. TSG: Tumor suppressor gene; UTR: Untranslated region.

Supplementary Figure 4

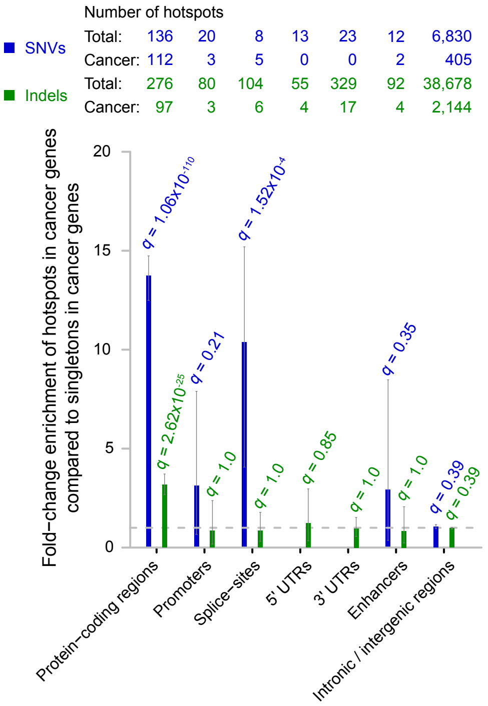

Supplementary Figure 4 | Fold-change enrichment of hotspots in cancer genes. Enrichment relative to the proportion of singletons in cancer genes; the proportion of singletons in cancer genes depends on the genomic region; numbers above bars are the total number and number of cancer hotspots in each region; error bars are Clopper-Pearson 95% confidence interval approximations. UTR: Untranslated region.

Supplementary Figure 5

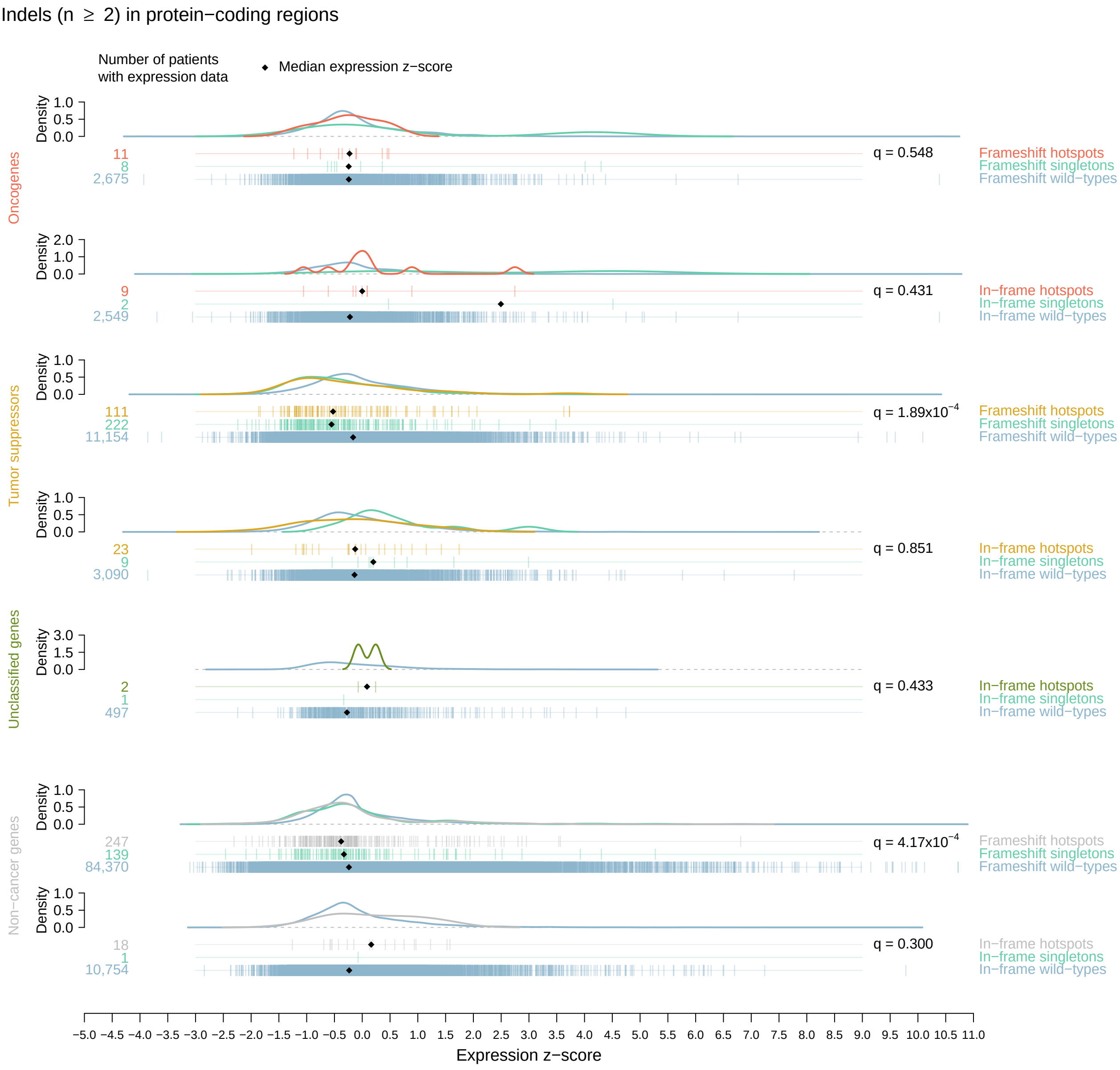

Supplementary Figure 5 | Distribution of expression z−scores for patients with protein−coding indel hotspots. Expression distributions for indel hotspots with two or more mutations in protein−coding regions of oncogenes, tumor suppressors, unclassified genes, and non−cancer genes divided into missense and nonsense mutations; singleton expression and wild−type expression for genes included in hotspot sets are shown directly below each set.

Supplementary Figure 6

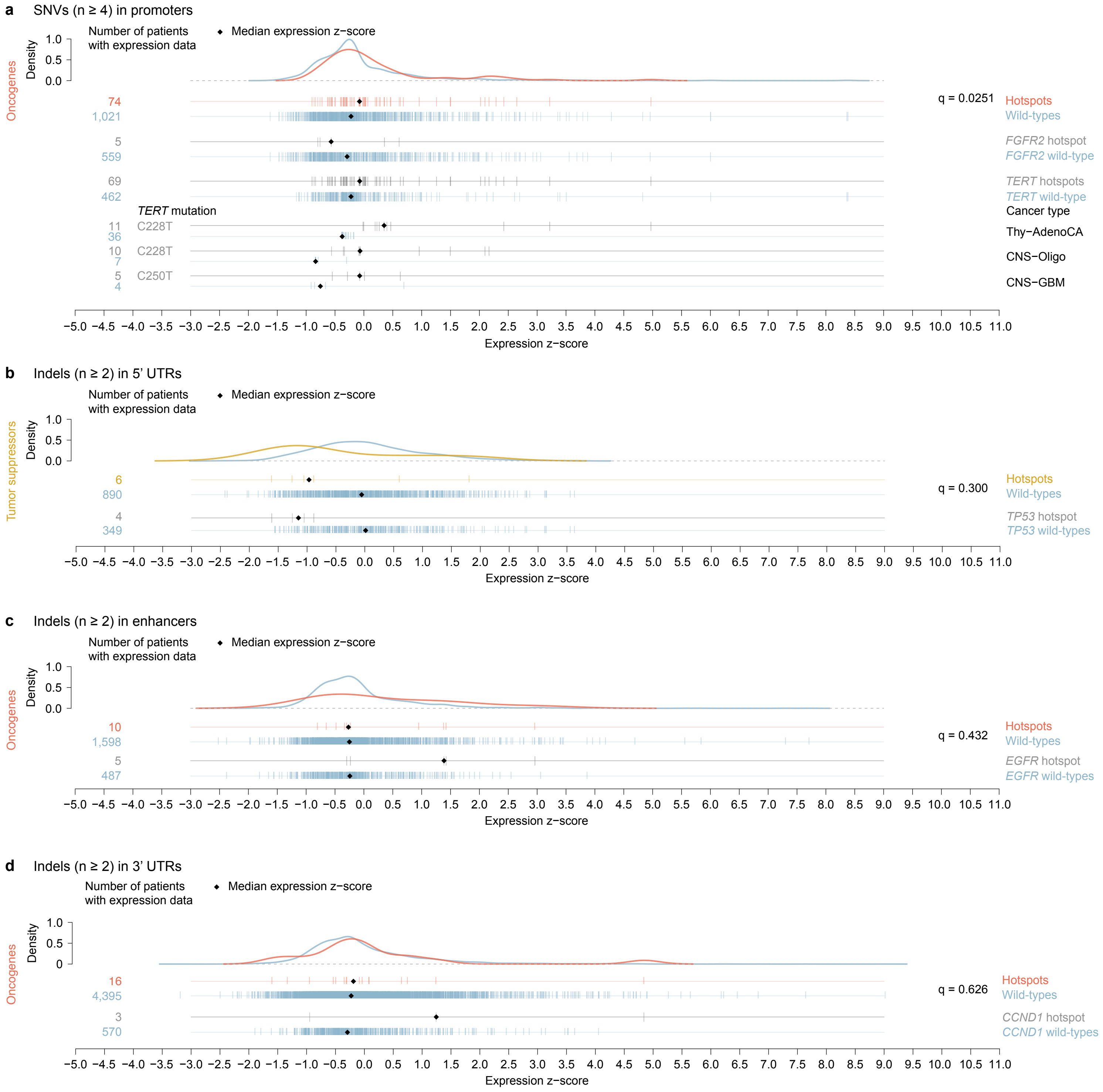

Supplementary Figure 6 | Distribution of expression z-scores for patients with hotspot mutations. a. Distribution for SNV hotspots in promoters of oncogenes including a zoom-in on the hotspot in *FGFR2*, and the two *TERT* hotspots; below is a stratification of *TERT* expression values per cancer type for three cohorts. **b.** Distribution for indel hotspots in 5' UTRs for tumor suppressors including a zoom-in on the *TP53* hotspot. **c.** Distribution for indel hotspots in enhancers for oncogenes including a zoom-in on the *EGFR* hotspot. **d.** Distribution for indel hotspots in 3' UTRs for oncogenes including a zoom-in on the *CCND1* hotspot. Remaining region / gene-type combinations are included in **Supplementary Figures 8** and **9**. CNS-GBM: Glioblastoma; CNS-Oligo: Oligodendroglioma; Thy-AdenoCA: Thyroid adenocarcinoma; UTR: Untranslated region.

Supplementary Figure 7

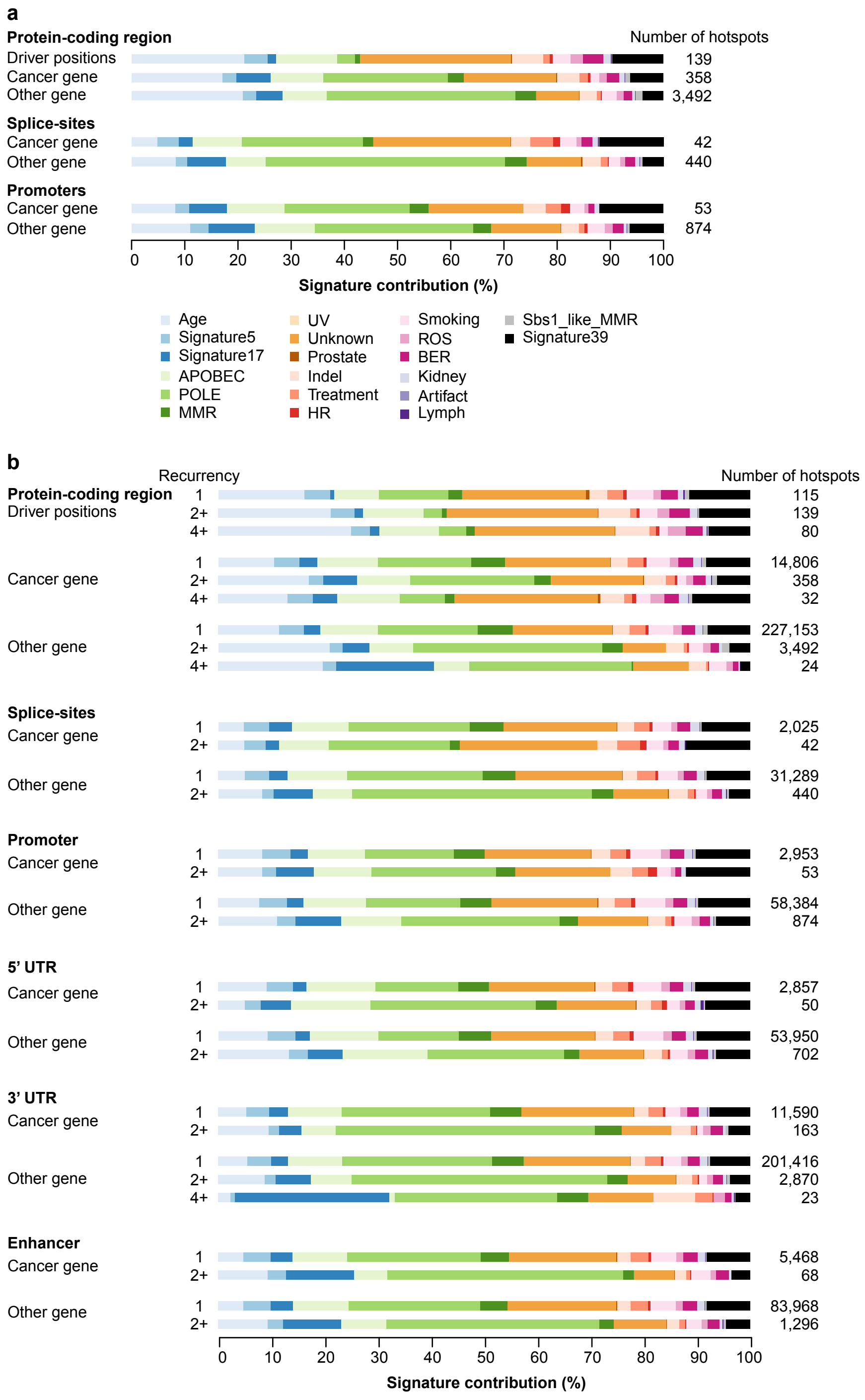

Supplementary Figure 7 | Signature contributions among hotspots. a. Signature contributions among hotspots in the protein-coding region, promoters, and splice-sites. The three upper rows show signature contributions among hotspots in the protein-coding region of i) hotspots in known driver positions, ii) hotspots in cancer genes, excluding driver positions, and iii) hotspots in other genes; the two middle rows show signature contributions among splice-sites of i) hotspots in cancer genes, and ii) hotspots in other genes; the two lower rows show signature contributions among promoter hotspots in i) cancer genes, and ii) other genes; all groups include hotspots with two or more mutations. Colors correspond to a specific signature or cluster of signatures. **b.** Signature contributions among hotspots across all regions. For each region, the hotspots are divided into known drivers (only protein-coding region), cancer genes, and other genes; each region-gene-type combination has a row for singletons (recurrency: 1); for all hotspots (recurrency: 2+) and for hotspots with four or more mutations (recurrency: 4+; for regions with at least 20 hotspots in this category). Colors correspond to a specific signature or cluster of signatures. APOBEC: Apolipoprotein B mRNA Editing Catalytic Polypeptide-like; POLE: polymerase ε; MMR: mismatch repair; UV: ultraviolet light; HR: homologous recombination; ROS: reactive oxygen species; BER: base excision repair; UTR: Untranslated region.

Supplementary Figure 8

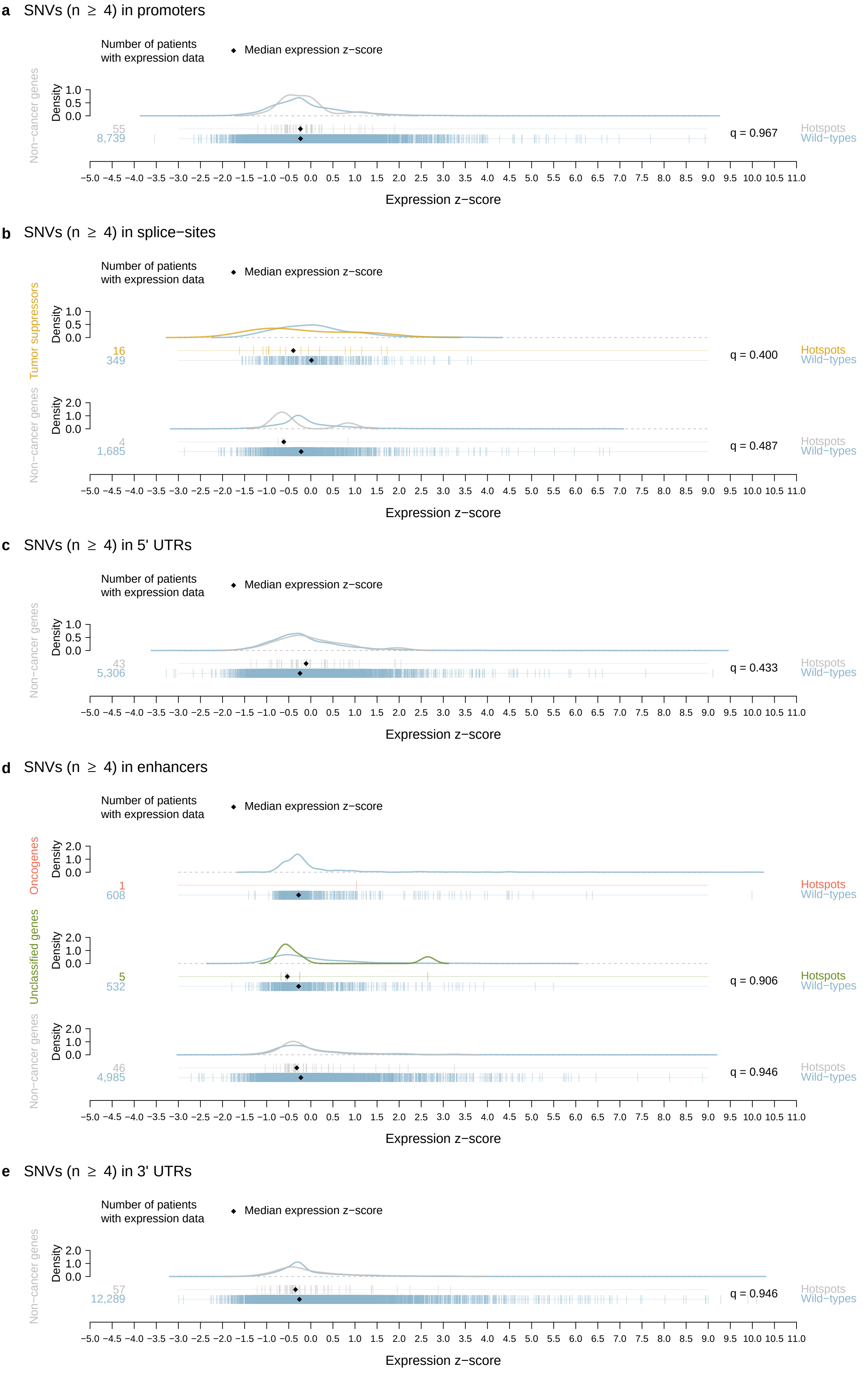

Supplementary Figure 8 | Distribution of expression z−scores for patients with SNV hotspots. a−e. Distribution for SNV hotspots in promoters of non−cancer genes (a), splice−sites of tumor suppressors and non−cancer genes (b), 5´ UTRs of non−cancer genes (c), enhancers of oncogenes, unclassified genes and non−cancer genes (d), and 3´ UTRs of non−cancer genes (e). UTR: Untranslated region.

Supplementary Figure 9

a Indels (n ≥ 2) in promoters

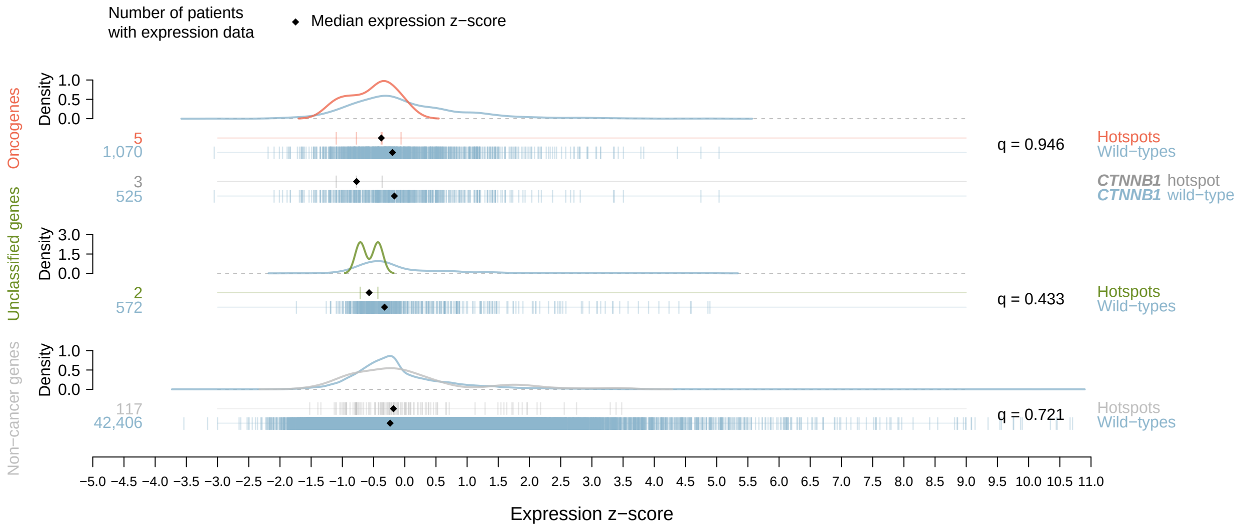

b Indels (n ≥ 2) in splice−sites

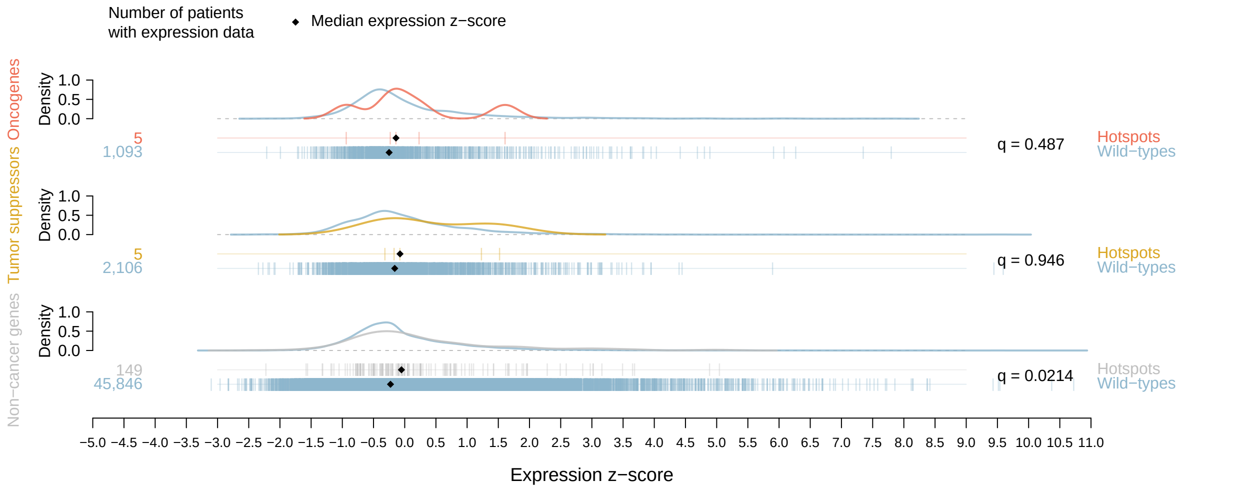

c Indels (n ≥ 2) in 5' UTRs

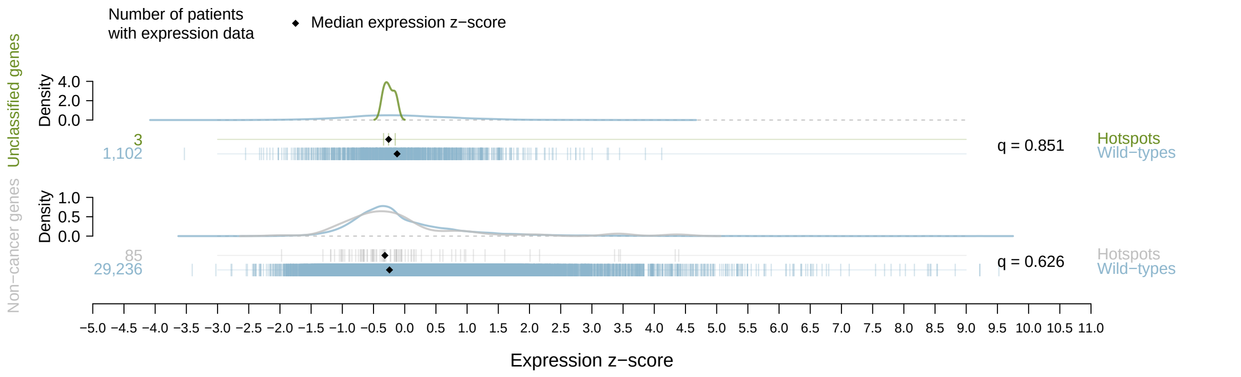

d Indels (n ≥ 2) in enhancers

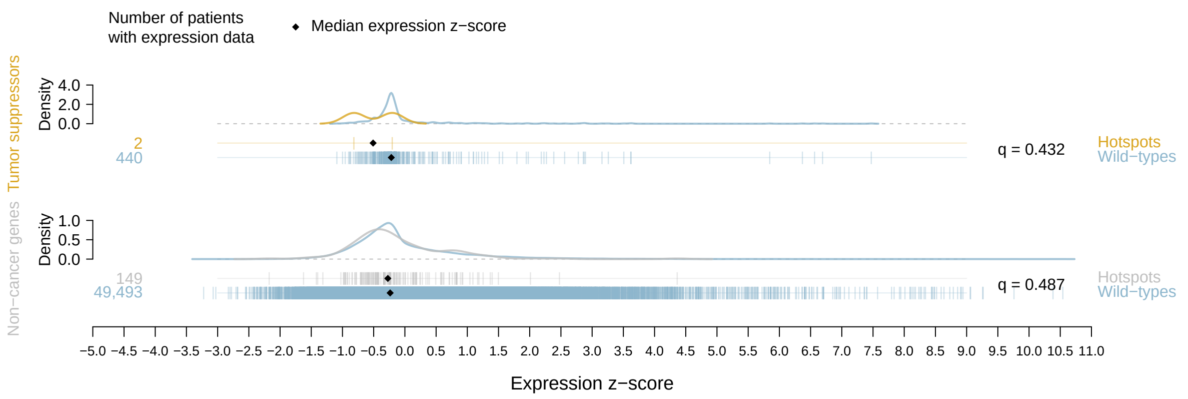

e Indels (n ≥ 2) in 3' UTRs

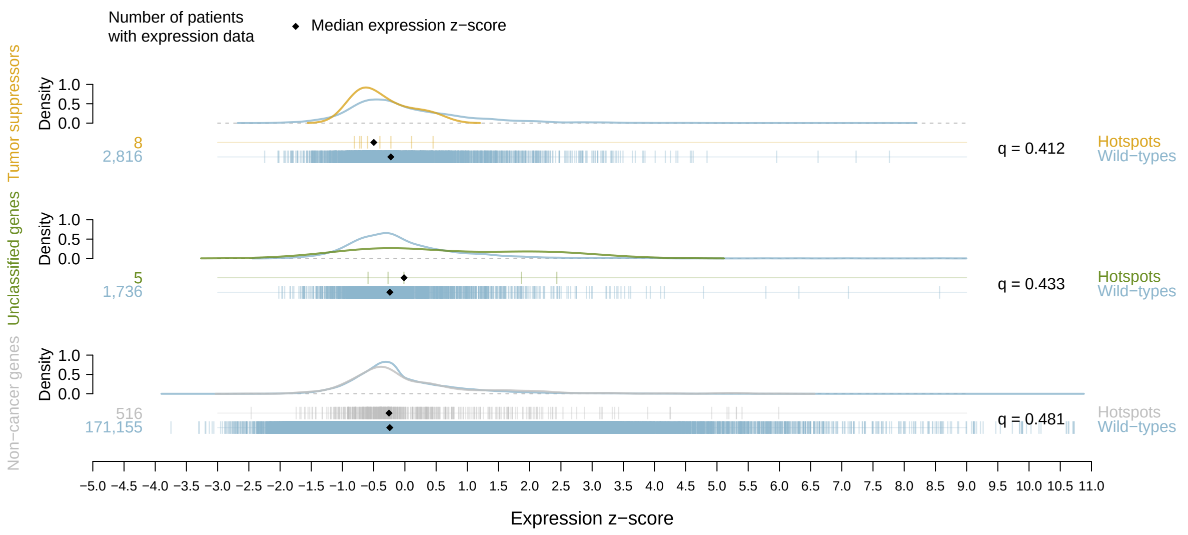

Supplementary Figure 9 | Distribution of expression z−scores for patients with indel hotspots. a−e. Distribution for indel hotspots in promoters of oncogenes, unclassified genes and non−cancer genes (a), splice−sites of oncogenes, tumor suppressors and non−cancer genes (b), 5´ UTRs of unclassified genes and non−cancer genes (c), enhancers of tumor suppressors and non−cancer genes (d), and 3´ UTRs of tumor suppressors, unclassified genes and non−cancer genes (e). UTR: Untranslated region.

Supplementary Table 1 | Significance evaluation of expectations under null-models (q-values)

SNVs

**Mutations at position**	0	1	2	3	4	5	6	7	8	9	10	11	12	13	14	15
**PSS[1] model**	1	0.01093	0.00011	1.41×10^{-6}	2.14×10^{-8}	3.78×10^{-10}	5.19×10^{-12}	7.49×10^{-14}	9.96×10^{-16}	1.21×10^{-17}	1.35×10^{-21}	1.37×10^{-21}	1.28×10^{-23}	1.11×10^{-25}	8.86×10^{-28}	6.63×10^{-30}
**Binomial model**	1	0.00108	5.83×10^{-5}	2.10×10^{-7}	5.69×10^{-10}	1.23×10^{-12}	2.22×10^{-15}	3.43×10^{-18}	4.63×10^{-21}	5.56×10^{-24}	6.00×10^{-27}	5.89×10^{-30}	5.29×10^{-33}	4.39×10^{-36}	3.38×10^{-39}	2.43×10^{-42}
**Mutations at position**	16	17	18	19	20	21	22	23	24	25	26	27	28	29	30	31
**PSS[1] model**	4.64×10^{-32}	3.06×10^{-34}	1.91×10^{-36}	1.13×10^{-38}	6.35×10^{-41}	3.41×10^{-43}	1.75×10^{-45}	8.59×10^{-48}	4.05×10^{-50}	1.84×10^{-52}	8.01×10^{-55}	3.37×10^{-57}	1.37×10^{-59}	5.35×10^{-62}	2.02×10^{-64}	7.41×10^{-67}
**Binomial model**	1.64×10^{-45}	1.04×10^{-48}	6.19×10^{-52}	3.50×10^{-55}	1.88×10^{-58}	9.64×10^{-62}	4.71×10^{-65}	2.20×10^{-68}	9.82×10^{-72}	4.22×10^{-75}	1.74×10^{-78}	6.90×10^{-82}	2.64×10^{-85}	9.75×10^{-89}	3.48×10^{-92}	1.20×10^{-95}
**Mutations at position**	32	33	34	35	36	37	38	39	40	41	42	43	44	45	46	47
**PSS[1] model**	2.62×10^{-69}	9.00×10^{-74}	2.99×10^{-74}	9.66×10^{-77}	3.03×10^{-79}	9.22×10^{-82}	2.73×10^{-84}	7.85×10^{-87}	2.20×10^{-89}	6.00×10^{-92}	1.59×10^{-94}	4.13×10^{-97}	1.04×10^{-99}	2.58×10^{-102}	6.21×10^{-105}	1.46×10^{-107}
**Binomial model**	4.01×10^{-99}	1.30×10^{-102}	4.08×10^{-106}	1.25×10^{-109}	3.70×10^{-113}	1.07×10^{-116}	2.99×10^{-120}	8.18×10^{-124}	2.18×10^{-127}	5.66×10^{-131}	1.43×10^{-134}	3.55×10^{-138}	8.58×10^{-142}	2.03×10^{-145}	4.69×10^{-149}	1.06×10^{-152}
**Mutations at position**	48	49	50	51	52	53	54	55	56	57	58	59	60	61	62	63
**PSS[1] model**	3.37×10^{-110}	7.58×10^{-133}	1.67×10^{-115}	3.60×10^{-118}	7.59×10^{-121}	1.57×10^{-123}	3.17×10^{-126}	6.30×10^{-129}	1.23×10^{-131}	2.34×10^{-134}	4.39×10^{-137}	8.07×10^{-140}	1.46×10^{-142}	2.58×10^{-145}	4.50×10^{-148}	7.70×10^{-151}
**Binomial model**	2.34×10^{-156}	5.07×10^{-160}	1.08×10^{-163}	2.24×10^{-167}	4.56×10^{-171}	9.12×10^{-175}	1.79×10^{-178}	3.44×10^{-182}	6.50×10^{-186}	1.21×10^{-189}	2.20×10^{-193}	3.94×10^{-197}	6.93×10^{-201}	1.20×10^{-204}	2.04×10^{-208}	3.42×10^{-212}
**Mutations at position**	64	65	66	67	68	69	70	71	72	73	74	75	76	77	78	79
**PSS[1] model**	1.30×10^{-153}	2.15×10^{-156}	3.49×10^{-159}	5.59×10^{-162}	8.80×10^{-165}	1.36×10^{-167}	2.08×10^{-170}	3.13×10^{-173}	4.63×10^{-176}	6.75×10^{-179}	9.70×10^{-182}	1.37×10^{-184}	1.92×10^{-187}	2.64×10^{-190}	3.58×10^{-193}	4.78×10^{-196}
**Binomial model**	5.63×10^{-216}	9.12×10^{-220}	1.46×10^{-223}	2.29×10^{-227}	3.54×10^{-231}	5.40×10^{-235}	8.11×10^{-239}	1.20×10^{-242}	1.75×10^{-246}	2.52×10^{-250}	3.57×10^{-254}	4.99×10^{-258}	6.89×10^{-262}	9.38×10^{-266}	1.26×10^{-269}	1.67×10^{-273}
**Mutations at position**	80	81	82	83	84	85	86	87	88	89	90	91	92	93	94	95
**PSS[1] model**	6.31×10^{-199}	8.22×10^{-202}	1.06×10^{-204}	1.34×10^{-207}	1.68×10^{-210}	2.07×10^{-213}	2.52×10^{-216}	3.04×10^{-219}	3.62×10^{-222}	4.25×10^{-225}	4.94×10^{-228}	5.67×10^{-231}	6.43×10^{-234}	7.20×10^{-237}	7.98×10^{-240}	8.74×10^{-243}
**Binomial model**	2.18×10^{-277}	2.82×10^{-281}	3.60×10^{-285}	4.53×10^{-289}	5.63×10^{-293}	6.92×10^{-297}	8.40×10^{-301}	1.01×10^{-304}	1.19×10^{-308}	1.40×10^{-312}	1.62×10^{-316}	1.85×10^{-320}	0	0	0	0
**Mutations at position**	96	97	98	99	100	101	102	103	104	105	106	107	108	109	110	111
**PSS[1] model**	9.47×10^{-246}	1.01×10^{-248}	1.07×10^{-251}	1.12×10^{-254}	1.17×10^{-257}	1.20×10^{-260}	1.21×10^{-263}	1.22×10^{-266}	1.21×10^{-269}	1.19×10^{-272}	1.16×10^{-275}	1.11×10^{-278}	1.06×10^{-281}	1.00×10^{-284}	9.40×10^{-288}	8.71×10^{-291}
**Binomial model**	0	0	0	0	0	0	0	0	0	0	0	0	0	0	0	0
**Mutations at position**	112	113	114	115	116	117	118	119	120	121	122-165					
**PSS[1] model**	7.99×10^{-294}	7.26×10^{-297}	6.54×10^{-300}	5.83×10^{-303}	5.15×10^{-306}	4.51×10^{-309}	3.92×10^{-312}	3.37×10^{-315}	2.87×10^{-318}	2.43×10^{-321}	0					
**Binomial model**	0	0	0	0	0	0	0	0	0	0	0					

Insertions

**Mutations at position**	0	1	2	3	4	5	6	7	8	9	10	11
**Binomial model**	1	2.54×10^{-4}	3.23×10^{-8}	2.74×10^{-16}	8.84×10^{-21}	3.72×10^{-25}	3.72×10^{-25}	1.36×10^{-29}	4.30×10^{-34}	1.21×10^{-38}	3.07×10^{-43}	7.07×10^{-48}

Deletions

**Mutations at position**	0	1	2-27
**Binomial model**	1	1	1.08×10^{-4}

[1] PSS: Position- and sample-specific

Supplementary Table 2 | Significance evaluation of expression abbreviations (q-values)

	Region	Cancer gene status	Classification	Difference in median (hotspot - WT)	q-value	Difference in median (singleton - WT)
SNVs (n ≥ 4)	Protein-coding regions	Oncogenes	Missense	0.25	**$1.77x10^{-6}$**	0.086
		TSGs[1]	Missense	0.28	**$1.77x10^{-6}$**	0.21
			Nonsense	-0.51	**$3.59x10^{-4}$**	-0.27
		Non-cancer	Missense	0.10	0.300	0.037
			Nonsense	-0.014	0.626	-0.18
	Promoters	Oncogenes		0.18	**0.0251**	
		Non-cancer		-0.0034	0.967	
	Splice-sites	TSGs[1]		-0.30	0.400	
		Non-cancer		-0.30	0.487	
	5' UTRs[2]	Non-cancer		0.13	0.433	
	Enhancers	Unclassified		-0.063	0.906	
		Non-cancer		0.0091	0.946	
	3' UTRs[2]	Non-cancer		-0.0082	0.946	
Indels (n ≥ 2)	Protein-coding regions	Oncogenes	In-frame	0.23	0.431	2.08
			Frameshift	-0.065	0.548	0.18
		TSGs[1]	In-frame	-0.11	0.851	0.55
			Frameshift	-0.36	**$1.89x10^{-4}$**	-0.40
		Unclassified	In-frame	0.35	0.433	-0.057
		Non-cancer	In-frame	0.29	0.300	0.16
			Frameshift	-0.17	**$4.17x10^{-4}$**	-0.15
	Promoters	Oncogenes		-0.38	0.946	
		Unclassified		-0.29	0.433	
		Non-cancer		0.037	0.721	
	Splice-sites	Oncogenes		0.14	0.487	
		TSGs[1]		0.38	0.946	
		Non-cancer		0.19	**0.0214**	
	5' UTRs[2]	TSGs[1]		-0.66	0.300	
		Unclassified		-0.13	0.851	
		Non-cancer		-0.053	0.626	
	Enhancers	Oncogenes		0.21	0.432	
		TSGs[1]		-0.39	0.432	
		Non-cancer		-0.049	0.487	
	3' UTRs[2]	Oncogenes		0.021	0.626	
		TSGs[1]		-0.22	0.412	
		Unclassified		0.46	0.433	
		Non-cancer		0.033	0.481	

[1] TSG: tumor suppressor gene; [2] UTR: untranslated region
