# Supplementary material for: The landscape and driver potential of site-specific hotspots across cancer genomes
Source: NPJ Genom Med. 2021 May 13;6:33. doi: 10.1038/s41525-021-00197-6 (PMC8119706; doi:10.1038/s41525-021-00197-6)
Supplement: Supplementary file 5 — Reporting Summary [file 41525_2021_197_MOESM5_ESM.pdf]

nature research

Corresponding author(s): Randi Istrup Juul, Jakob Skou Pedersen

Last updated by author(s): Apr 8, 2021

Reporting Summary

Nature Research wishes to improve the reproducibility of the work that we publish. This form provides structure for consistency and transparency in reporting. For further information on Nature Research policies, see our Editorial Policies and the Editorial Policy Checklist.

Statistics

For all statistical analyses, confirm that the following items are present in the figure legend, table legend, main text, or Methods section.

n/a	Confirmed	
☐	☒	The exact sample size (*n*) for each experimental group/condition, given as a discrete number and unit of measurement
☐	☒	A statement on whether measurements were taken from distinct samples or whether the same sample was measured repeatedly
☐	☒	The statistical test(s) used AND whether they are one- or two-sided<br>*Only common tests should be described solely by name; describe more complex techniques in the Methods section.*
☒	☐	A description of all covariates tested
☐	☒	A description of any assumptions or corrections, such as tests of normality and adjustment for multiple comparisons
☐	☒	A full description of the statistical parameters including central tendency (e.g. means) or other basic estimates (e.g. regression coefficient) AND variation (e.g. standard deviation) or associated estimates of uncertainty (e.g. confidence intervals)
☐	☒	For null hypothesis testing, the test statistic (e.g. *F*, *t*, *r*) with confidence intervals, effect sizes, degrees of freedom and *P* value noted<br>*Give P values as exact values whenever suitable.*
☒	☐	For Bayesian analysis, information on the choice of priors and Markov chain Monte Carlo settings
☒	☐	For hierarchical and complex designs, identification of the appropriate level for tests and full reporting of outcomes
☐	☒	Estimates of effect sizes (e.g. Cohen's *d*, Pearson's *r*), indicating how they were calculated

Our web collection on statistics for biologists contains articles on many of the points above.

Software and code

Policy information about availability of computer code

Data collection	All data collection was done by the PCAWG consortium as described in the marker paper (ICGC/TCGA Pan-Cancer Analysis of Whole Genomes Consortium, Nature 2020)
Data analysis	The following software was used for data analysis: R version 3.5.1; bedtools version 2.29.2; ucsc-overlapselect version 366. Besides using base R, we further used the R packages IRanges, dplyr and metap.

For manuscripts utilizing custom algorithms or software that are central to the research but not yet described in published literature, software must be made available to editors and reviewers. We strongly encourage code deposition in a community repository (e.g. GitHub). See the Nature Research guidelines for submitting code & software for further information.

Data

Policy information about availability of data

All manuscripts must include a data availability statement. This statement should provide the following information, where applicable:

- Accession codes, unique identifiers, or web links for publicly available datasets
- A list of figures that have associated raw data
- A description of any restrictions on data availability

The dataset used for this study can be downloaded from the ICGC Data Portal under the PCAWG release: https://dcc.icgc.org/releases/PCAWG. Additional information on accessing the data, can be found at https://docs.icgc.org/pcawg/data/. Most of the data is publicly available, but all the TCGA data and the potentially identifiable data from ICGC is protected and needs permission to download. To access the protected data, researches will need to apply to the TCGA Data Access Committee (DAC) via dbGaP (https://dbgap.ncbi.nlm.nih.gov/aa/wga.cgi?page=login), and the ICGC Data Access Compliance Office (DACO; http://icgc.org/daco), respectively.

Field-specific reporting

Please select the one below that is the best fit for your research. If you are not sure, read the appropriate sections before making your selection.

☒ Life sciences ☐ Behavioural & social sciences ☐ Ecological, evolutionary & environmental sciences

For a reference copy of the document with all sections, see nature.com/documents/nr-reporting-summary-flat.pdf

Life sciences study design

All studies must disclose on these points even when the disclosure is negative.

Sample size	The data was provided by the PCAWG consortium. We use the 2,583 white-listed samples from the PCAWG project. We did not perform statistical calculation on sample size.
Data exclusions	The data was provided by the PCAWG consortium. We excluded the 304 patients with skin cancers or lymphoid malignanicies (PCAWG cohorts: Skin-Melanoma, Lymph-BNHL, Lymph-CLL, and Lymph-NOS) as these cancer types have highly active mutational processes leading to excess amounts of hotspot mutations, potentially diluting pan-cancer driver signals. We further excluded the X and Y chromosomes as they harbored more artifactual mutation calls.
Replication	The data was provided by the PCAWG consortium. This is a descriptive / exploratory study.
Randomization	The data was provided by the PCAWG consortium. Individual participants was not grouped.
Blinding	The data was provided by the PCAWG consortium. Individual participants was not grouped.

Reporting for specific materials, systems and methods

We require information from authors about some types of materials, experimental systems and methods used in many studies. Here, indicate whether each material, system or method listed is relevant to your study. If you are not sure if a list item applies to your research, read the appropriate section before selecting a response.

Materials & experimental systems

n/a	Involved in the study	
☒	☐	Antibodies
☒	☐	Eukaryotic cell lines
☒	☐	Palaeontology and archaeology
☒	☐	Animals and other organisms
☒	☐	Human research participants
☒	☐	Clinical data
☒	☐	Dual use research of concern

Methods

n/a	Involved in the study	
☒	☐	ChIP-seq
☒	☐	Flow cytometry
☒	☐	MRI-based neuroimaging
